# ﻿Infraspecific variation of some brown *Parmeliae* (in Poland) – a comparison of ITS rDNA and non-molecular characters

**DOI:** 10.3897/mycokeys.85.70552

**Published:** 2021-12-22

**Authors:** Katarzyna Szczepańska, Beata Guzow-Krzemińska, Jacek Urbaniak

**Affiliations:** 1 Department of Botany and Plant Ecology, Wrocław University of Environmental and Life Sciences, pl. Grunwaldzki 24a, PL-50-363 Wrocław, Poland Wrocław University of Environmental and Life Sciences Wrocław Poland; 2 Department of Plant Taxonomy and Nature Conservation, Faculty of Biology, University of Gdańsk, Wita Stwosza 59, PL-80-308 Gdańsk, Poland University of Gdańsk Gdańsk Poland

**Keywords:** Cryptic species, haplotype, lichenised fungi, Parmeliaceae, phylogeny, taxonomy

## Abstract

Infraspecific variation of the ITS rDNA region of some brown *Parmeliae* occurring in Poland is studied and compared with non-molecular characters. Haplotype networks are used to illustrate the variability within the species. Both newly-produced sequences from Central Europe and from all over the world, downloaded from the GenBank, are used.

The number of haplotypes found for each taxon ranged from five in *Melaneliastygia* to 12 in *Melaneliahepatizon* and *Montaneliadisjuncta*; however, their numbers correlate with the number of specimens tested. New haplotypes for *Melaneliaagnata*, *M.hepatizon* and *Cetrariacommixta* are found. Based on our 169-sample dataset, we could not infer any geographical correlation, either locally or world-wide. Many of the analysed haplotypes were widely distributed and the same haplotype was often shared between temperate and polar populations. A comparison of molecular, morphological, anatomical and chemical characters also shows no correlation.

## ﻿Introduction

The brown *Parmeliae* (Esslinger 1977) have been an object of numerous studies (Guzow-Krzemińska and Węgrzyn 2003; Blanco et al. 2005; Crespo et al. 2010, 2011; Nelsen et al. 2011; Divakar et al. 2012; Thell et al. 2012; Leavitt et al. 2014, 2015) and, due to this exceptional attention, they are one of the best-studied assemblages in the family Parmeliaceae. These lichens are a polyphyletic group possessing foliose, a dark to medium brown thallus and usually lacking atranorin or usnic acid in the cortex (Esslinger 1977; Blanco et al. 2004).

For many years, one of the largest genera within this group was *Melanelia* Essl., segregated from *Parmelia* Ach. by Esslinger (1978) to accommodate species with brown, foliose thalli and an N– cortex layer. However, during the following years, it has been demonstrated that the genus *Melanelia* s. lat. was polyphyletic and several new genera were distinguished within it, such as *Melanelixia* O. Blanco et al., *Melanohalea* O. Blanco et al. (Blanco et al. 2004) and *Montanelia* Divakar et al. (Divakar et al. 2012). In traditional terms, brown *Parmeliae* includes other genera, such as *Allantoparmelia* (Vain.) Essl., *Pleurosticta* Petr. and some species of *Xanthoparmelia* (Vain.) Hale. Moreover, due to the historical taxonomic approach (Thell 1995; Rico et al. 2005) and the similarity in the morphological and anatomical features of thalli, *Cetrariacommixta* is also referred to this group.

Our studies have focused on the saxicolous species of *Melanelia* and *Montanelia* genera. According to Otte et al. (2005), species of these genera are arctic-alpine, circumpolar and occur on silicate rocks in the mountain areas of the Northern Hemisphere, including Arctic Regions (Divakar et al. 2012). Nowadays, *Melanelia* s. str. is restricted to a small clade of saxicolous, cetrarioid lichens and includes four species: *M.agnata* (Nyl.) A. Thell, *M.hepatizon* (Ach.) A. Thell, *M.pseudoglabra* (Essl.) Essl. and *M.stygia* (L.) Essl. According to Thell (1995), these species are characterised by broadly clavate asci with a small tholus and a broad axial body, a thick, paraplectenchymatous cortex and dumb-bell-shaped pycnoconidia. *Montanelia*, representing the parmelioid clade, includes eight species: *M.disjuncta* (Erichsen) Divakar, A. Crespo, Wedin & Essl., *M.occultipanniformis* S.D. Leav., Essl., Divakar, A. Crespo & Lumbsch, *M.panniformis* (Nyl.) Divakar, A. Crespo, Wedin & Essl., *M.predisjuncta* (Essl.) Divakar, A. Crespo, Wedin & Essl., *M.saximontana* (R.A. Anderson & W.A. Weber) S.D. Leav., Essl., Divakar, A. Crespo & Lumbsch, *M.secwepemc* S.D. Leav., Essl., Divakar, A. Crespo & Lumbsch, *M.sorediata* (Ach.) Divakar, A. Crespo, Wedin & Essl. and *M.tominii* (Oxner) Divakar, A. Crespo, Wedin & Essl. (Divakar et al. 2012; Leavitt et al. 2015; Leavitt et al. 2016). The characteristic features of the *Montanelia* genus are short and narrow lobes, with flat to convex lobe margins, a non-pored epicortex, cylindrical to fusiform conidia, a medulla containing orcinol depsides and flat, effigurate pseudocyphellae (absent only in *M.sorediata*; Divakar et al. 2012). Three of these species (*M.disjuncta*, *M.panniformis* and *M.sorediata*) have broad, intercontinental distributions, with no evidence of phylogeographic substructure (Leavitt et al. 2015).

The genera *Melanelia* and *Montanelia* have been the subject of a critical revision in Poland and data concerning their distribution, ecology and morphological, anatomical and chemical features are presented in previous papers (Szczepańska et al. 2015; Szczepańska and Kossowska 2017). However, recent molecular studies imply that both genera may include previously unrecognised species-level diversity (Divakar et al. 2012; Leavitt et al. 2014), especially within Icelandic populations of *M.stygia* (Xu et al. 2017).

One of the goals of this study was to assess the intraspecific internal transcribed spacer (ITS) rDNA variability in brown *Parmeliae* species. Investigations of genetic variation in lichen-forming symbionts have advanced considerably in recent years and resulted in interesting conclusions (Palice and Printzen 2004; Lindblom and Ekman 2006; Domaschke et al. 2012; Starosta and Svoboda 2020). Although brown *Parmeliae* appear to be well studied in taxonomic terms, there are insufficient molecular data to estimate their genetic variation. Most of the available data concern samples collected in a few regions of the world, such as Europe and North America. The North American species of this group were studied in Greenland and Canada (Leavitt et al. 2014; Leavitt et al. 2015), while samples from Europe originated mainly from the north – Iceland, Finland, Norway and Sweden (Blanco et al. 2004; Divakar et al. 2012; Xu et al. 2017). Therefore, we decided to fill in the gap in sampling and focused our study on samples collected in Central Europe. We have used phylogenetic trees and haplotype networks to investigate the extent of molecular differences between newly-generated sequences from samples collected in Central Europe (Austria, Czech Republic, Germany, Poland and Slovakia) and others originating from different geographical regions. Due to additional samplings from previously unexplored areas, it was possible to evaluate and compare the genetic variability of the studied specimens in Central Europe with samples from other regions of the world and to identify areas with the greatest haplotype diversity. In addition, we analysed morphological, anatomical and chemical characters of collected specimens to find a potential correlation between phenotypic characters and genetic variation of the studied taxa. By analysing genetic diversity and geographical distribution of identified haplotypes, as well as phenotypic characters of collected samples, we tried to better define and designate the species boundaries within analysed taxa. Special emphasis was placed on analysis of European, Greenlandic and Icelandic samples of *M.agnata* and *M.stygia* to revise the hypothesis assuming a semi-cryptic or cryptic nature of their potential species-level diversity.

## ﻿Materials and methods

### ﻿Taxon sampling

The study is based on collections from the AMNH, C and WRSL Herbaria, as well as the private material of Dr Maria Kossowska (hb. Kossowska). Our sampling focused on saxicolous representatives of the Parmeliaceae family occurring in Poland, with brown, foliose thalli, such as *Cetrariacommixta*, *Melaneliaagnata*, *M.hepatizon*, *M.stygia*, *Montaneliadisjuncta* and *M.sorediata*. We also included the holotype of *Melaneliaagnata* (*Platysmaagnatum*; Austria, Tirol, Gerölle unter dem Gneissfelsen zum wilden see. Auf dem Kraxentrag, Tirol, Brenner 225, Aug 1871, H-NYL 36086), borrowed from Herbarium of W. Nylander in Helsinki in our analyses.

Specimens for molecular study were selected after detailed morphological and chemical analyses. Due to DNA degradation, it was not possible to use samples collected more than three years prior to the DNA extraction procedure in most cases. As the *Melaneliaagnata* and *M.stygia* specimens from Greenland and Iceland were collected more than 10 years ago, we had to limit our phylogenetic analyses to ITS rDNA markers and used the sequences stored in GenBank. Before phylogenetic analysis, newly-obtained ITS rDNA sequences were subjected to a BLAST search (Altschul et al. 1997). The final ITS dataset used in this study includes 52 sequences newly generated and 117 sequences downloaded from GenBank (Table 1).

**Table 1:** . The species and specimens used in the phylogenetic analyses and/or haplotype network analyses, sequences newly generated for this study are in bold.

Species	Year of collection	Isolate	Locality	Collector (-s)	Voucher specimens (herbarium)	GenBank no. (ITS)
***Cetrariellacommixta***	**2007**	**36**	**Poland, Sudety Mts**	**Kossowska, M.**	**Kossowska 107 (personal herbarium)**	**MZ029708**
***Cetrariellacommixta***	**2008**	**37**	**Poland, Sudety Mts**	**Kossowska, M.**	**Kossowska 231 (personal herbarium)**	**MZ029709**
***Cetrariellacommixta***	**2016**	**97**	**Poland, Sudety Mts**	**Szczepańska, K.**	**Szczepańska 1137 (WRSL)**	**MZ029733**
***Cetrariellacommixta***	**2016**	**124**	**Poland, Sudety Mts**	**Szczepańska, K.**	**Szczepańska 1184 (WRSL)**	**MZ029753**
***Cetrariellacommixta***	**2018**	**129**	**Germany, Bayerischer Wald**	**Szczepańska, K.**	**Szczepańska 1267 (WRSL)**	**MZ029758**
*Cetrariellacommixta*			Finland	Haikonen, V.	Haikonen 19093 (H)	AF451796
*Cetrariellacommixta*	1996		Canada, British Columbia	Miao, V. & Taylor, T.		AF451797
*Cetrariellacommixta*			Sweden	Wedin, M.	Wedin 8143 (UPS)	GU994554
*Cetrariellacommixta*			Spain, Segovia	Rico, V. J.	15555 (MAF)	GU994555
*Cetrariellacommixta*	2004	CCO 01	Sweden, Lule Lappmark		1273926 (LD)	KC990132
*Cetrariellacommixta*		6543	Greenland, SEm, Tasilaq	Hansen, E. S.	Hansen ESH-10B.139 (C)	KF257934
*Cetrariellacommixta*		6547	Greenland, SWm, Qeqertaq	Hansen, E. S.	Hansen ESH-09.087 (C)	KF257935
*Cetrariellacommixta*		6567	Greenland, S, Igaliku	Hansen, E. S.	Hansen ESH-08.173 (C)	KF257936
*Cetrariellacommixta*		6570	Greenland, SWm, Midgard	Hansen, E. S.	Hansen ES-09.030 (C)	KF257937
*Cetrariellacommixta*		6572	Greenland, S, Aappilattoq	Hansen, E. S.	Hansen ES-04.070 (C)	KF257938
*Cetrariellacommixta*		6573	Greenland, SWm, Qeqertaq	Hansen, E. S.	Hansen ES-09.064 (C)	KF257939
*Cetrariellacommixta*	2014		Norway, Finnmark	Westberg, M.	O-L-195926	KY266843
***Melaneliaagnata***	**2016**	**102**	**Poland, Karpaty Mts**	**Szczepańska, K.**	**Szczepańska 1151 (WRSL)**	**MZ029737**
***Melaneliaagnata***	**2016**	**103**	**Poland, Karpaty Mts**	**Szczepańska, K.**	**Szczepańska 1150 (WRSL)**	**MZ029738**
*Melaneliaagnata*	2009	6549	Greenland, SW m, Jensens Nunatakker	Hansen, E. S.	Hansen ESH-09.478 (C)	KF257940
*Melaneliaagnata*	2009	6553	Greenland, SW m, Jensens Nunatakker	Hansen, E. S.	Hansen ESH-09.435 (C)	KF257941
*Melaneliaagnata*	2007	6563	Greenland, N, Constable Bugt	Hansen, E. S.	Hansen ESH-07.464 (C)	KF257942
*Melaneliaagnata*	2002	MX_MS2	Iceland, Imi	Heiðmarsson, S.	LA29683 (AMHN)	KY508672
*Melaneliaagnata*	2005	MX_MS3	Iceland, Ino	Kristinsson, H.	LA27562 (AMHN)	KY963373
*Melaneliaagnata*	2008	MX_MS4	Iceland, Isu	Hjaltadóttir, A.	LA30974 (AMHN)	KY508673
*Melaneliaagnata*	2012	MX_MS5	Iceland, Ino	Heiðmarsson, S.	LA31859 (AMHN)	KY963374
*Melaneliaagnata*	2014		Norway, Sor-Trondelag	Timdal, E.	O-L-196376	MK812394
*Melaneliaculbersonii*			USA	Lendemer, J.	Lendemer 13821 (NY)	KR995286
***Melaneliahepatizon***	**2016**	**83**	**Poland, Sudety Mts**	**Szczepańska, K.**	**Szczepańska 1051 (WRSL)**	**MZ029723**
***Melaneliahepatizon***	**2016**	**91**	**Poland, Sudety Mts**	**Szczepańska, K.**	**Szczepańska 1120 (WRSL)**	**MZ029717**
***Melaneliahepatizon***	**2016**	**95**	**Poland, Sudety Mts**	**Szczepańska, K.**	**Szczepańska 1136A (WRSL)**	**MZ029731**
***Melaneliahepatizon***	**2016**	**96**	**Poland, Sudety Mts**	**Szczepańska, K.**	**Szczepańska 1136B (WRSL)**	**MZ029732**
***Melaneliahepatizon***	**2016**	**98**	**Poland, Sudety Mts**	**Szczepańska, K.**	**Szczepańska 1138 (WRSL)**	**MZ029734**
***Melaneliahepatizon***	**2016**	**109**	**Poland, Karpaty Mts**	**Szczepańska, K.**	**Szczepańska 1153 (WRSL)**	**MZ029741**
***Melaneliahepatizon***	**2016**	**110**	**Poland, Karpaty Mts**	**Szczepańska, K.**	**Szczepańska 1154A (WRSL)**	**MZ029730**
***Melaneliahepatizon***	**2016**	**111**	**Poland, Karpaty Mts**	**Szczepańska, K.**	**Szczepańska 1154B (WRSL)**	**MZ029743**
***Melaneliahepatizon***	**2016**	**113**	**Poland, Karpaty Mts**	**Szczepańska, K.**	**Szczepańska 1144 (WRSL)**	**MZ029745**
***Melaneliahepatizon***	**2016**	**116**	**Slovakia, Karpaty Mts**	**Szczepańska, K.**	**Szczepańska 1146 (WRSL)**	**MZ029746**
***Melaneliahepatizon***	**2016**	**117**	**Slovakia, Karpaty Mts**	**Szczepańska, K.**	**Szczepańska 1147 (WRSL)**	**MZ029747**
***Melaneliahepatizon***	**2016**	**119**	**Poland, Sudety Mts**	**Szczepańska, K.**	**Szczepańska 1180 (WRSL)**	**MZ029748**
***Melaneliahepatizon***	**2016**	**122**	**Poland, Sudety Mts**	**Szczepańska, K.**	**Szczepańska 1182 (WRSL)**	**MZ029751**
***Melaneliahepatizon***	**2018**	**128**	**Germany, Bayerischer Wald**	**Szczepańska, K.**	**Szczepańska 1269 (WRSL)**	**MZ029757**
*Melaneliahepatizon*	1996		Canada, British Columbia		Thell & Veer BC-9677 (LD)	AF141369
*Melaneliahepatizon*	2001	DNA-AT934	Italy, Trentino-Alto Adige (south Tirolia)	Feuerer T. & Thell A. s. n.	LD, HBG	AF451776
*Melaneliahepatizon*			Sweden	Wedin, M.	Wedin 6812 (UPS)	DQ980016
*Melaneliahepatizon*			Greenland, NWn, Siorapuluk	Hansen, E. S.	Hansen ESH-09B.164 (C)	KF257943
*Melaneliahepatizon*			Greenland, NWn, Qaanaaq	Hansen, E. S.	Hansen ESH-09B.026 (C)	KF257944
*Melaneliahepatizon*			Greenland, SEm, Tasilaq	Hansen, E. S.	Hansen ESH-10B.014 (C)	KF257945
*Melaneliahepatizon*			Greenland, SWm, Nuuq	Hansen, E. S.	Hansen ESH-10A.019 (C)	KF257946
*Melaneliahepatizon*			Greenland, S, Qaqortoq	Hansen, E. S.	Hansen ESH-08.036 (C)	KF257947
*Melaneliahepatizon*			Greenland, S, Igaliku	Hansen, E. S.	Hansen ESH-08.170 (C)	KF257948
*Melaneliahepatizon*			Greenland, S, Narssarsuag	Hansen, E. S.	Hansen ESH-08.263 (C)	KF257949
*Melaneliahepatizon*			Greenland, S, Igaliku	Hansen, E. S.	Hansen ESH-08.215 (C)	KF257950
*Melaneliahepatizon*			Greenland, SWm, Midgard	Hansen, E. S.	Hansen ESH-09.386 (C)	KF257951
*Melaneliahepatizon*			Greenland, SWm, Frederikshab Isblink	Hansen, E. S.	Hansen ESH-09.324 (C)	KF257952
*Melaneliahepatizon*			Greenland, S, Igaliku	Hansen, E. S.	Hansen ESH-08.477 (C)	KF257953
*Melaneliahepatizon*	2014		Norway, Finnmark	Westberg, M.	O-L-195864	KY266879
*Melaneliahepatizon*	2003	MH1	Iceland, IAu		LA30501 (AMHN)	KY508674
*Melaneliahepatizon*	2007	MH3	Iceland, IVe		LA30676 (AMHN)	KY508675
*Melaneliahepatizon*	2007	MH4	Iceland, IVe		LA30674 (AMHN)	KY508676
*Melaneliahepatizon*	2007	MH5	Iceland, IVe		LA30675 (AMHN)	KY508677
*Melaneliahepatizon*	2007	MH6	Iceland, IVe		LA30673 (AMHN)	KY508678
*Melaneliahepatizon*	2014	MH9	Iceland, INo		LA20781 (AMHN)	KY508679
*Melaneliahepatizon*	2013	MH10	Iceland, INv		LA30117 (AMHN)	KY508680
*Melaneliahepatizon*	2012	MH11	Iceland, Inv		LA31861 (AMHN)	KY963376
*Melaneliahepatizon*	2014		Norway, Hordaland	Timdal, E.	O-L-195807	MK812512
*Melaneliahepatizon*	2015		Norway, Nord-Trondelag	Bendiksby, M. et al.	O-L-201254	MK812070
*Melaneliahepatizon*	2013		Norway, Buskerud	Rui, S. & Timdal, E.	O-L-184723	MK812188
***Melaneliastygia***	**2007**	**40**	**Poland, Sudety Mts**	**Kossowska, M.**	**Kossowska 123 (personal herbarium)**	**MZ029710**
***Melaneliastygia***	**2009**	**42**	**Austria, Tyrol**	**Szczepańska, K.**	**Szczepańska 737 (WRSL)**	**MZ029712**
***Melaneliastygia***	**2016**	**94**	**Poland, Sudety Mts**	**Szczepańska, K.**	**Szczepańska 1134 (WRSL)**	**MZ029719**
***Melaneliastygia***	**2016**	**104**	**Poland, Karpaty Mts**	**Szczepańska, K.**	**Szczepańska 1152 (WRSL)**	**MZ029739**
***Melaneliastygia***	**2016**	**108**	**Poland, Karpaty Mts**	**Szczepańska, K.**	**Szczepańska 1149 (WRSL)**	**MZ029740**
***Melaneliastygia***	**2016**	**112**	**Poland, Karpaty Mts**	**Szczepańska, K.**	**Szczepańska 1160 (WRSL)**	**MZ029744**
***Melaneliastygia***	**2018**	**127**	**Czech Republic, Šumava**	**Szczepańska, K.**	**Szczepańska 1265 (WRSL)**	**MZ029756**
*Melaneliastygia*			Finland, Nyland	Kuusinen, M.	FIN-9714 (LD)	AF115763
*Melaneliastygia*			Italy	Feurerer, T & Thell, A.	DNA-AT922 (LD)	AF451775
*Melaneliastygia*			Finland, Enonkoski	Haikonen, V.	Haikonen 20365	AY611097
*Melaneliastygia*			Austria, Steiermark	Hafellner, J.	Hafellner 51658	AY611121
*Melaneliastygia*	2008	6551	Greenland, S, Qaqortoq	Hansen, E. S.	Hansen ESH-08.036 (C)	KF257954
*Melaneliastygia*	2008	6569	Greenland, S, Igaliku	Hansen, E. S.	Hansen ESH-08.478 (C)	KF257955
*Melaneliastygia*	1998	MX_MS1	Iceland, IAu	Kristinsson, H.	LA19972 (AMHN)	KY508681
*Melaneliastygia*	2014	MX_MS3	Iceland, IAu	Kristinsson, H.	LA20775 (AMHN)	KY508682
*Melaneliastygia*	2013	MX_MS4	Iceland, IAu	Kristinsson, H.	LA16894 (AMHN)	KY508683
*Melaneliastygia*	2000	MX_MS2	Iceland, IAu	Kristinsson, H.	LA28243 (AMHN)	KY963375
*Melaneliastygia*	2013		Norway, Buskerud	Rui, S. & Timdal, E.	O-L-184736	MK812608
*Melaneliastygia*	2014		Norway, Sor-Trondelag	Timdal, E.	O-L-196377	MK812312
***Montaneliadisjuncta***	**2013**	**50**	**Poland, Sudsty Forelands**	**Szczepańska, K.**	**Szczepańska 969 (WRSL)**	**MZ029713**
***Montaneliadisjuncta***	**2014**	**51**	**Poland, Sudety Mts**	**Szczepańska, K.**	**Szczepańska 989 (WRSL)**	**MZ029714**
***Montaneliadisjuncta***	**2015**	**57**	**Poland, Sudety Foothills**	**Szczepańska, K.**	**Szczepańska 1023 (WRSL)**	**MZ029715**
***Montaneliadisjuncta***	**2015**	**78**	**Poland, Sudety Mts**	**Szczepańska, K.**	**Szczepańska 1034 (WRSL)**	**MZ029716**
***Montaneliadisjuncta***	**2015**	**79**	**Poland, Sudety Mts**	**Szczepańska, K.**	**Szczepańska 1038 (WRSL)**	**MZ029711**
***Montaneliadisjuncta***	**2015**	**80**	**Poland, Sudety Mts**	**Szczepańska, K.**	**Szczepańska 1039 (WRSL)**	**MZ029720**
***Montaneliadisjuncta***	**2016**	**81**	**Poland, Sudety Mts**	**Szczepańska, K.**	**Szczepańska 1047 (WRSL)**	**MZ029721**
***Montaneliadisjuncta***	**2016**	**82**	**Poland, Sudety Mts**	**Szczepańska, K.**	**Szczepańska 1048 (WRSL)**	**MZ029722**
***Montaneliadisjuncta***	**2016**	**85**	**Poland, Sudety Mts**	**Szczepańska, K.**	**Szczepańska 1054 (WRSL)**	**MZ029724**
***Montaneliadisjuncta***	**2016**	**86**	**Poland, Sudety Mts**	**Szczepańska, K.**	**Szczepańska 1081 (WRSL)**	**MZ029725**
***Montaneliadisjuncta***	**2016**	**87**	**Poland, Sudety Mts**	**Szczepańska, K.**	**Szczepańska 1082 (WRSL)**	**MZ029726**
***Montaneliadisjuncta***	**2016**	**88**	**Poland, Sudety Mts**	**Szczepańska, K.**	**Szczepańska 1110 (WRSL)**	**MZ029727**
***Montaneliadisjuncta***	**2016**	**89**	**Poland, Sudety Mts**	**Szczepańska, K.**	**Szczepańska 1111 (WRSL)**	**MZ029728**
***Montaneliadisjuncta***	**2016**	**90**	**Poland, Sudety Mts**	**Szczepańska, K.**	**Szczepańska 1119 (WRSL)**	**MZ029729**
***Montaneliadisjuncta***	**2016**	**92**	**Pland, Sudety Foothils**	**Szczepańska, K.**	**Szczepańska 1127 (WRSL)**	**MZ029755**
***Montaneliadisjuncta***	**2016**	**93**	**Pland, Sudety Foothils**	**Szczepańska, K.**	**Szczepańska 1128 (WRSL)**	**MZ029718**
***Montaneliadisjuncta***	**2016**	**120**	**Poland, Sudety Mts**	**Szczepańska, K.**	**Szczepańska 1181A (WRSL)**	**MZ029749**
***Montaneliadisjuncta***	**2016**	**121**	**Poland, Sudety Mts**	**Szczepańska, K.**	**Szczepańska 1181B (WRSL)**	**MZ029750**
***Montaneliadisjuncta***	**2016**	**123**	**Poland, Sudety Mts**	**Szczepańska, K.**	**Szczepańska 1183 (WRSL)**	**MZ029752**
***Montaneliadisjuncta***	**2016**	**125**	**Poland, Sudety Mts**	**Szczepańska, K.**	**Szczepańska 1185 (WRSL)**	**MZ029754**
***Montaneliadisjuncta***	**2016**	**126**	**Poland, Sudety Mts**	**Szczepańska, K.**	**Szczepańska 1230 (WRSL)**	**MZ029742**
***Montaneliadisjuncta***	**2018**	**130**	**Czech Republic, Šumava**	**Szczepańska, K.**	**Szczepańska 1271 (WRSL)**	**MZ029759**
*Montaneliadisjuncta*			Austria, Steiermark		Mayrhofer 13743	AY611077
*Montaneliadisjuncta*			India		MAF-Lich 15512	GU994556
*Montaneliadisjuncta*			United Kingdom		Coppins 637	JX974654
*Montaneliadisjuncta*			Greenland, NWn, Siorapaluk	Hansen, E. S.	Hansen ESH-09B.363 (C)	KF257957
*Montaneliadisjuncta*		3921	Canada, Yukon Territory	Spribille, T.	Spribille s.n.	KP771824
*Montaneliadisjuncta*		3963	Greenland, Northwest	Hansen, E. S.	Hansen ESH-09B.051 (C)	KP771827
*Montaneliadisjuncta*		3995	USA, Maine	Harris, R.	Harris 52938 (NY)	KP771828
*Montaneliadisjuncta*		4503	Norway, Tromso	Bjerke, J.W.	Bjerke WP286-2 (TLE)	KP771829
*Montaneliadisjuncta*		4851	Canada, Yukon Territory	Esslinger, T. L.	Esslinger BP94-3 (TLE)	KP771830
*Montaneliadisjuncta*		5970	USA, Alaska	Esslinger, T. L.	Esslinger 19403 (TLE)	KP771831
*Montaneliadisjuncta*		6575	Greenland, Northwest, Siorapaluk	Hansen, E. S.	Hansen ESH-09B.323 (C)	KP771833
*Montaneliadisjuncta*		MDISJUNCT	Sweden, Lycksele Lappmark	Wedin, M.	Wedin 7143 (UPS)	KP771834
*Montaneliadisjuncta*		MEDI637	United Kingdom, Scotland	Coppins, B.	Coppins s.n (MAF)	KP771835
*Montaneliadisjuncta*		MESO773	India, Uttaranchal	Divakar, P. K.	MAF-Lich 15512	KP771837
*Montaneliadisjuncta*	2014		Norway, Finnmark, Vadso	Haugan, R.	O-L-198675	KY266910
*Montaneliadisjuncta*	2007	MD8	Iceland, INo		LA30657 (AMHN)	KY508686
*Montaneliadisjuncta*			Sweden	Wedin, M.	Wedin 7143 (UPS)	DQ980015
*Montaneliadisjuncta*			USA	Lumbsch, H. T.	Lumbsch 2010/M7 (F)	JX126181
*Montaneliadisjuncta*			USA, Maine		Harris 55589 (NY)	KF257960
*Montaneliadisjuncta*			USA, Alaska		Esslinger 19403 (TLE)	KF257968
*Montaneliadisjuncta*			Canada		Goward 08	JX974658
*Montaneliadisjuncta*			Canada, Yukon		Spribille s.n. (GZU)	KF257956
*Montaneliadisjuncta*			Canada, Alberta		Holzinger 1061 (UBC)	KF257962
*Montaneliadisjuncta*			Canada, British Columbia		Esslinger BP109-1 (TLE)	KF257964
*Montaneliadisjuncta*			Canada, British Columbia		Esslinger BP97-01 (TLE)	KF257965
*Montaneliadisjuncta*			Canada, Yukon		Esslinger BP94-2 (TLE)	KF257966
*Montaneliadisjuncta*			Canada, Yukon		Esslinger BP94-3 (TLE)	KF257967
*Montaneliadisjuncta*			Canada, New Brunswick		McMullin 7483 (TLE)	KF257969
*Montaneliadisjuncta*			Canada, British Columbia		Goward 2008 (MAF)	KP771836
*Montaneliadisjuncta*			Greenland, S, Igaliku	Hansen, E. S.	Hansen ESH-08.304 (C)	KF257958
*Montaneliadisjuncta*			Greenland, NWn, Qaanaaq	Hansen, E. S.	Hansen ESH-09B.051 (C)	KF257959
*Montaneliadisjuncta*			Greenland, S, Igaliku	Hansen, E. S.	Hansen ESH-08.216 (C)	KF257970
*Montaneliadisjuncta*			Greenland, NWn, Siorapuluk	Hansen, E. S.	Hansen ESH-09B.323 (C)	KF257971
*Montaneliadisjuncta*		3956	Greenland, Northwest	Hansen, E. S.	Hansen ESH-09B.363 (C)	KP771825
*Montaneliadisjuncta*		3957	Greenland, South	Hansen, E. S.	Hansen ESH-08.304 (C)	KP771826
*Montaneliadisjuncta*		6574	Greenland, South, Igaliku	Hansen, E. S.	Hansen ESH-08.216 (C)	KP771832
*Montaneliadisjuncta*			Norway, Tromso		Bjerke WP286-2 (TLE)	KF257961
*Montaneliadisjuncta*			India, Uttar Pradesh		Divakar 15512 (MAF-Lich)	KF257972
*Montaneliadisjuncta*	2000	MD2	Iceland, Iau		LA28245 (AMHN)	KY963377
*Montaneliadisjuncta*	2009	MD5	Iceland, Ino		LA31552 (AMHN)	KY963378
*Montaneliadisjuncta*	2007	MD3	Iceland, Ino		LA30617 (AMHN)	KY508684
*Montaneliadisjuncta*			Canada, British Columbia		Goward 10-19 (UBC)	KF257963
*Montaneliadisjuncta*	2014		Norway, Sor-Trondelag	Timdal, E.	O-L-196357	MK811711
*Montaneliadisjuncta*	2014		Norway, Finnmark	Timdal, E.	O-L-195590	MK811852
*Montaneliadisjuncta*	2006	MD4	Iceland, Ino		LA27588	KY508685
***Montaneliasorediata***	**2016**	**100**	**Poland, Karpaty Mts**	**Szczepańska, K.**	**Szczepańska 1156 (WRSL)**	**MZ029735**
***Montaneliasorediata***	**2016**	**101**	**Poland, Karpaty Mts**	**Szczepańska, K.**	**Szczepańska 1155 (WRSL)**	**MZ029736**
*Montaneliasorediata*		4001	USA, Pennsylvania	Lendemer, J.	Lendemer 13329 (NY)	KF257978
*Montaneliasorediata*		4824	Canada, British Columbia	Esslinger, T.L.	Esslinger BP111-1 (TLE)	KF257979
*Montaneliasorediata*		4884	USA, Alaska	Esslinger, T.L.	Esslinger BP73-6 (TLE)	KF257980
*Montaneliasorediata*		5981	Russia, Khabarovskiy Krai	Spribille, T.	Spribille 31972 (GZU)	KF257981
*Montaneliasorediata*		6380	Canada, Ontario	McMullin, T.	McMullin 8139 (TLE)	KF257982
*Montaneliasorediata*		B_8600	Japan, Mt. Ohyama	Ohmura, Y.	Ohmura 9666 (TNS)	KM386101
*Montaneliasorediata*		MESO778	Sweden, Vasterbotten	Wedin, M.	Wedin 6862 (UPS)	KP771845
*Montaneliasorediata*		4001	USA, Pennsylvania	Lendemer, J.	Lendemer 13329 (NY)	KP771846
*Montaneliasorediata*		5981	Russia, Khabarovskiy Krai	Spribille, T.	Spribille 31972 (GZU)	KP771847
*Montaneliasorediata*	2014		Norway, Telemark	Timdal, E.	O-L-195791	MK811963
*Montaneliasorediata*	2014		Norway, Troms	Timdal, E.	O-L-195658	MK811965
*Montaneliasorediata*	2016		Norway, Buskerud	Dahl, M. S., Kistenich, S. D., Timdal, E., Toreskaas, A. K.	O-L-204941	MK811977
*Montaneliasorediata*		C_4670	Canada, British Columbia	Bjork, C.	Bjork 15153 (UBC)	KM386102

### ﻿Morphology and chemistry

The morphology and anatomy of the specimens were studied in detail with dissecting and light microscopes, following routine techniques. All specimens were examined for the assessment of the morphological characters, such as lobe width and morphology (flat/convex), the appearance of the upper surface (dull/glossy), the appearance of the lower surface (light/dark), apothecia morphology (sessile/constricted), appearance and position of pycnidia (marginal/laminal), appearance and position of the pseudocyphellae (marginal/laminal), size and shape of conidia (bacilliform/bifusiform), as well as ascospore size. For light microscopy, vertical sections of apothecia were cut by hand using a razor blade and mounted in water. Hymenium and conidia measurements were made in water and ascospore measurements were made in 10% potassium hydroxide (KOH). At least ten measurements of morphological variables and measurements of 20 spores and conidia were made for each sample and their minimum and maximum values were calculated.

The TLC analyses were undertaken in A and C solvent systems using the standardised method of Culberson (1972) and following Orange et al. (2001).

### ﻿DNA extraction, PCR amplification and DNA sequencing

Genomic DNA was extracted from specimens after cell disruption in a Mixer Mill MM400 (Retsch, Haan, Germany) using a CTAB method according to the standard protocol of isolation (Doyle and Doyle 1987). The quality of the isolated DNA was determined using 1% TBE agarose electrophoresis. PCR reactions were performed in 20 μl reaction tubes that contained a Dream Taq reaction buffer containing MgCl_2_, a 0.2 mM dNTP mix, 1u DreamTaq DNA polymerase (Thermo Fisher Scientific, Waltham, MA, USA), 0.5 mM each ITS1 and ITS4 primers and 0.8 μl of total genomic DNA. The adequate annealing temperature was determined using the gradient method. The PCR programme consisted of an initial denaturation at 95 °C for 6 min, according to a previous study (Szczepańska et al. 2020), followed by 30 cycles at 95 °C for 30 sec, 51.2 °C for 45 sec, 72 °C for 45 sec, with a final extension at 72 °C for 10 min. While performing PCR, the Veriti Thermal Cycler (Life Technologies, Carlsbad, CA, USA) was used. Amplification products were separated in 1% agarose gel, photographed and compared with the DNA mass ruler (Thermo Fisher Scientific Waltham, MA, USA). Bands corresponding to the ITS region were excised from the agarose gel and then purified by ethanol precipitation. Cleaned samples were sent to a sequencing service (Genomed, Warszawa, Poland). All laboratory analyses were performed at the Department of Botany and Plant Ecology at the Wrocław University of Environmental and Life Sciences.

### ﻿Sequence alignment and phylogenetic analysis

The newly-generated sequences and selected representatives of brown saxicolous Parmeliaceae were aligned using the Guidance 2 server (Landan and Graur 2008; Penn et al. 2010; Sela et al. 2015) employing the MAFFT algorithm (Katoh et al. 2002) followed by elimination of terminal ends. The final alignment consisted of 117 sequences of 535 sites. Further, we used Partition Finder 2 (Lanfear et al. 2016) implemented at the CIPRES Science Gateway (Miller et al. 2010). Two different models were found for partitions: GTR+G for ITS1 and ITS2 and K80+G for the 18S and 5.8S regions.

Moreover, phylogenetic analysis of all *Melanelia* sequences was also performed. Newly-generated sequences and these downloaded from GenBank, together with representatives of *Cetrariacommixta*, which were further used as an outgroup, were aligned using the Guidance 2 server (Landan and Graur 2008; Penn et al. 2010; Sela et al. 2015) employing the MAFFT algorithm (Katoh et al. 2002) followed by elimination of unreliable columns. The final alignment consisted of 76 sequences of 803 sites. Further, we used jModeltest 2.1 (Darriba et al. 2012) implemented at the CIPRES Science Gateway (Miller et al. 2010) and the K80+G model was selected.

Bayesian analysis was carried out using a Markov Chain Monte Carlo (MCMC) method, in MrBayes v. 3.2.6 (Huelsenbeck and Ronquist 2001; Ronquist and Huelsenbeck 2003) on the CIPRES Web Portal (Miller et al. 2010) using best models. Two parallel MCMC runs were performed, each using four independent chains and four million generations, sampling every 1000^th^ tree. Posterior probabilities (PP) were determined by calculating a majority-rule consensus tree after discarding the initial 25% trees of each chain as the burn-in.

A Maximum Likelihood (ML) analysis was performed using RAxML-HPC2 v.8.2.10 (Stamatakis 2014) with 1000 ML bootstrap iterations (BS) and the GTRGAMMAI model for both analyses. Phylogenetic trees were visualised using FigTree v. 1.4.2 (Rambaut 2012) and modified in Inkscape (https://inkscape.org/).

### ﻿Haplotype networks

Newly-generated sequences of the ITS rDNA marker, together with sequences downloaded from GenBank from specimens of *Cetrariacommixta*, *Melaneliaagnata*, *M.hepatizon*, *M.stygia*, *Montaneliadisjuncta* and *M.sorediata*, were aligned separately for each species using Seaview software (Galtier et al. 1996; Gouy et al. 2010). TCS networks (Clement et al. 2002) were created as implemented in PopART software (http://popart.otago.ac.nz). Nucleotide diversity per site was calculated using DnaSP v.6 software (Rozas et al. 2017).

## ﻿Results

### ﻿Phylogeny and haplotype networks

A total of 169 sequences were analysed in this study.

The RAxML tree did not contradict the Bayesian trees topologies for the strongly-supported branches and only the latter is shown with posterior probabilities. The bootstrap support values BS ≥ 70 and PP ≥ 0.95 were considered to be significant and are shown near the branches. In Fig. S1, three main, highly supported lineages representing *Melanelia* spp. (i.e. *M.agnata*, *M.hepatizon* and *M.stygia*), *Montanelia* spp. (i.e. *M.disjuncta* and *M.sorediata*) and *Cetrariacommixta* were distinguished. The newly-sequenced specimens clustered together with other representatives of the species downloaded from GenBank. Amongst them, *Melaneliastygia* is not monophyletic, but forms two separate well-supported clades.

**Figure 1.**
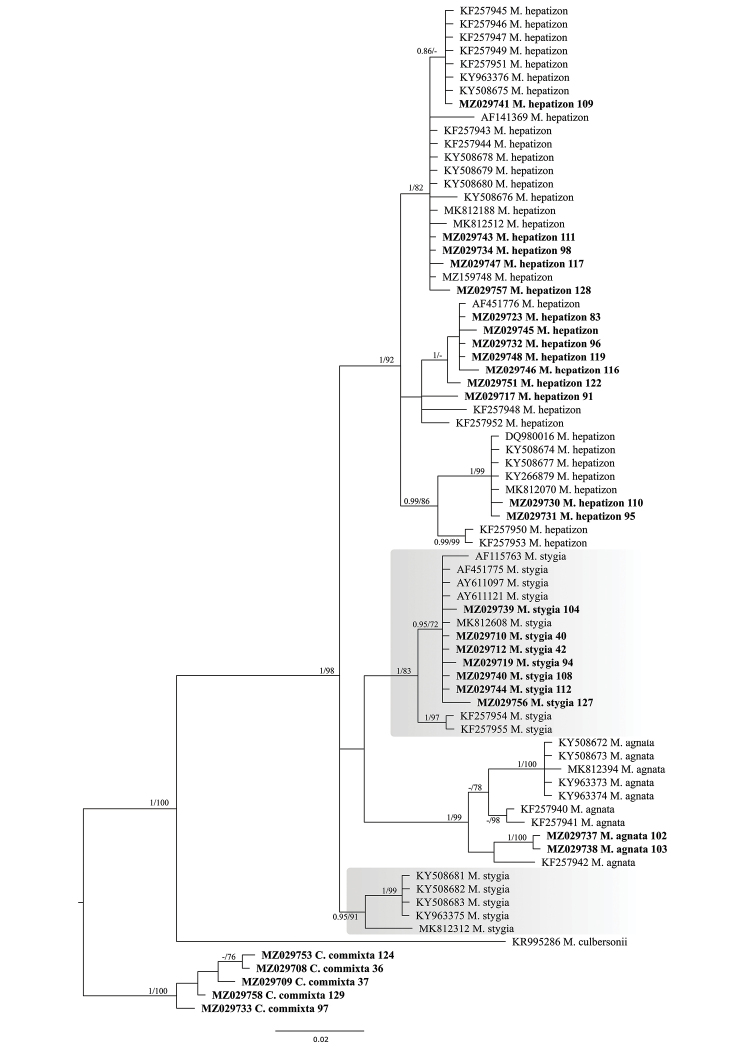
Phylogenetic relationships of *Melanelia* spp., based on Bayesian analysis of the ITS rDNA dataset. Posterior probabilities and Maximum Likelihood bootstrap values are shown near the internal branches. Newly-generated sequences are additionally described with isolate numbers following the species names and are marked in bold. GenBank accession numbers of sequences downloaded from GenBank are listed on the tree with species names.

Moreover, to better understand phylogenetic relationships in the *Melanelia*, we performed additional analysis for all available ITS rDNA sequences from representatives of this genus. The Bayesian tree is presented in Fig. 1 with posterior probabilities and the bootstrap support values presented near the branches and with *Cetrariacommixta* as an outgroup. In this tree, *Melaneliastygia* also forms two separate, highly-supported clades.

**Figure 2.**
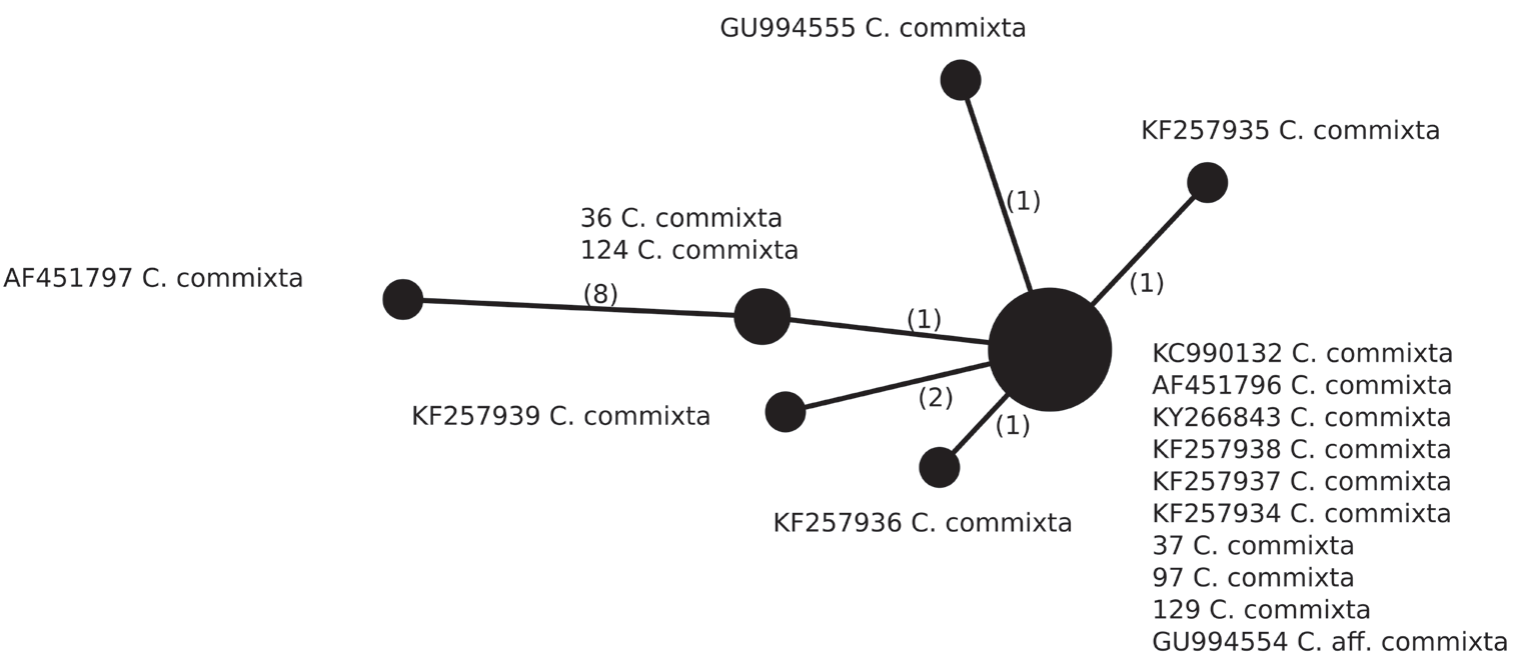
Haplotype network, based on ITS rDNA sequences from specimens of *Cetrariacommixta*. Newly-generated sequences are described with isolate numbers preceding the species names. Sequences downloaded from GenBank are described with their accession numbers. Mutational changes are presented as numbers in brackets near lines between haplotypes.

**Figure 3.**
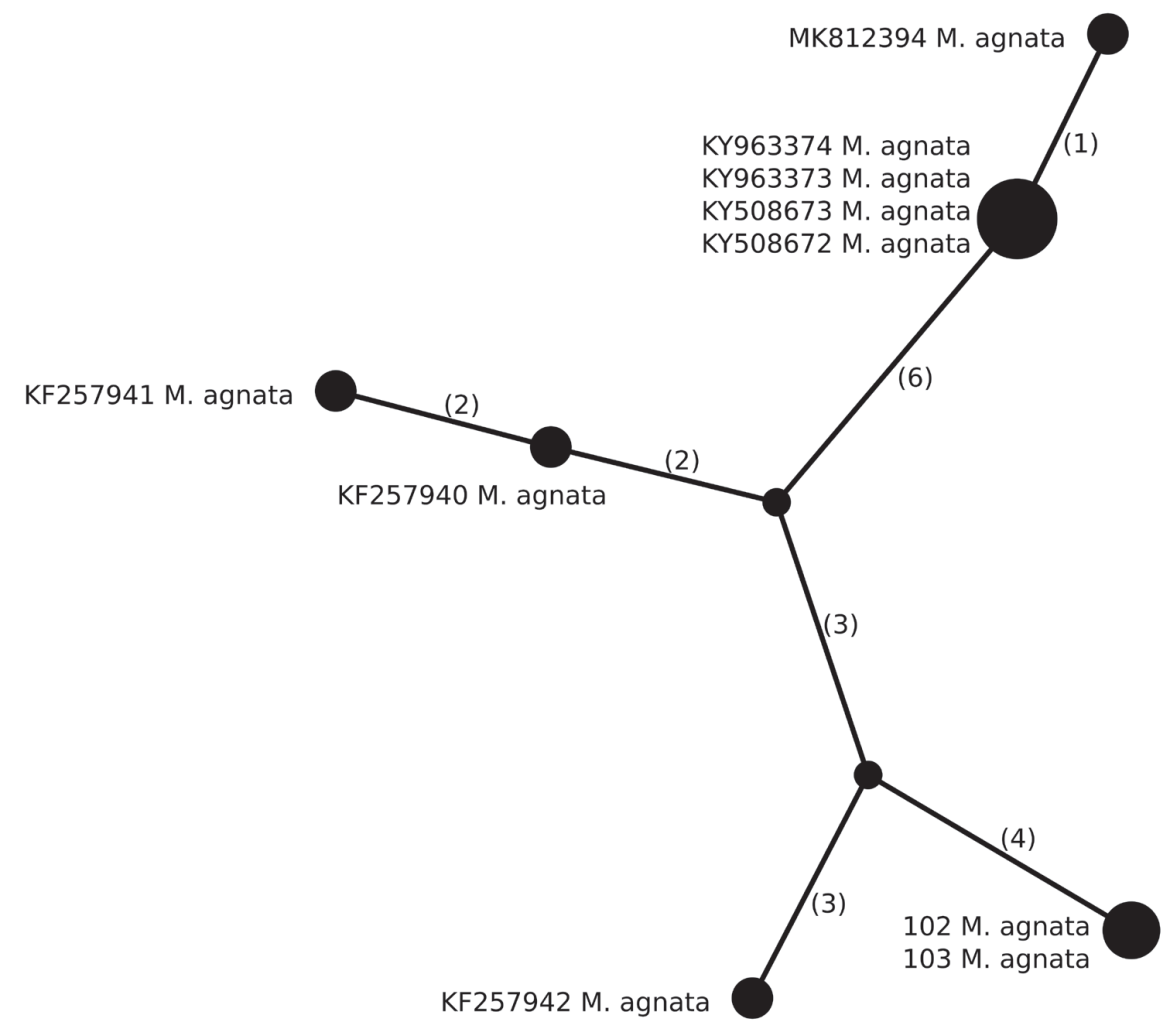
Haplotype network, based on ITS rDNA sequences from specimens of *Melaneliaagnata*. Newly-generated sequences are described with isolate numbers preceding the species names. Sequences downloaded from GenBank are described with their accession numbers. Mutational changes are presented as numbers in brackets near lines between haplotypes.

**Figure 4.**
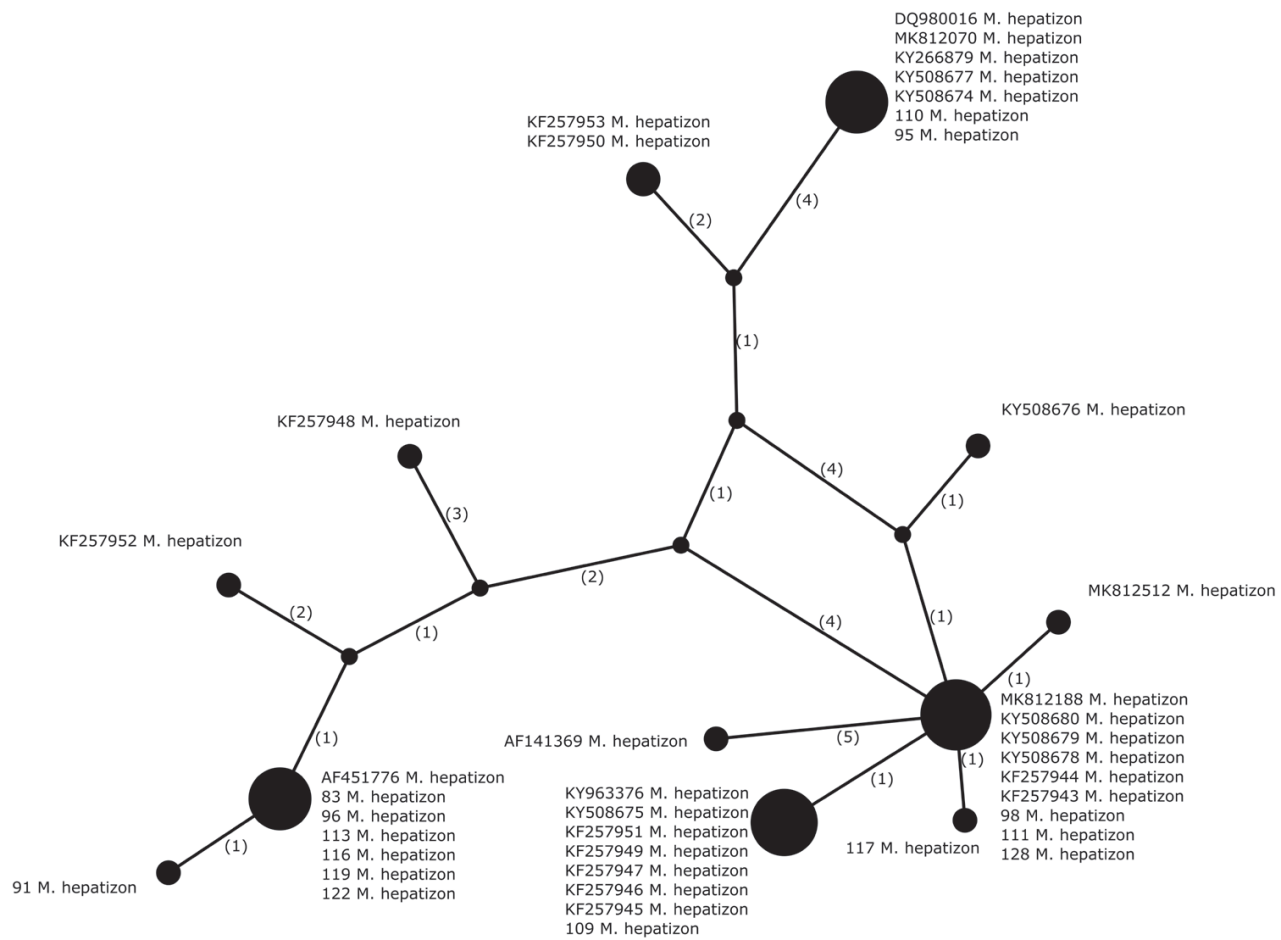
Haplotype network, based on ITS rDNA sequences from specimens of *Melaneliahepatizon*. Newly-generated sequences are described with isolate numbers preceding the species names. Sequences downloaded from GenBank are described with their accession numbers. Mutational changes are presented as numbers in brackets near lines between haplotypes.

**Figure 5.**
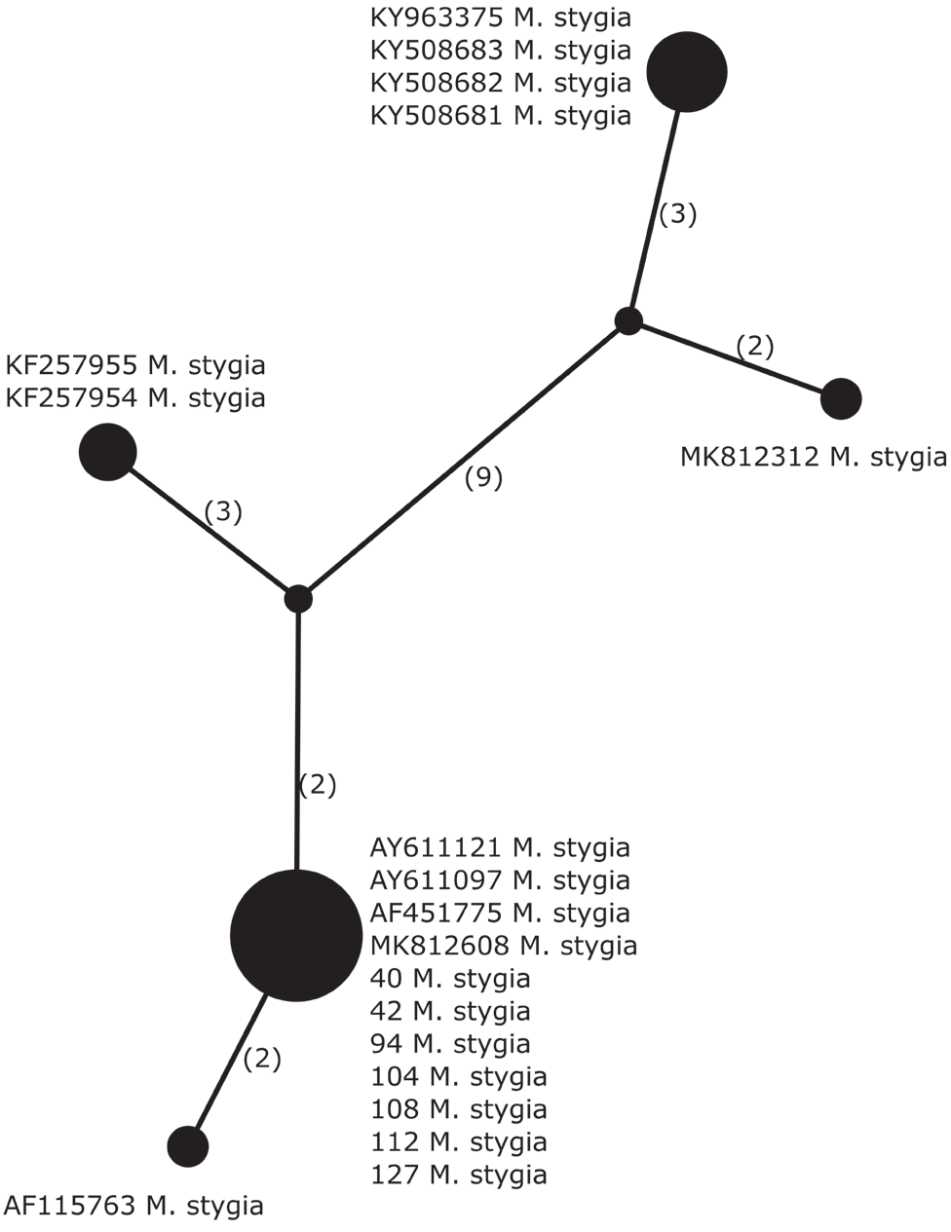
Haplotype network, based on ITS rDNA sequences from specimens of *Melaneliastygia*. Newly-generated sequences are described with isolate numbers preceding the species names. Sequences downloaded from GenBank are described with their accession numbers. Mutational changes are presented as numbers in brackets near lines between haplotypes.

**Figure 6.**
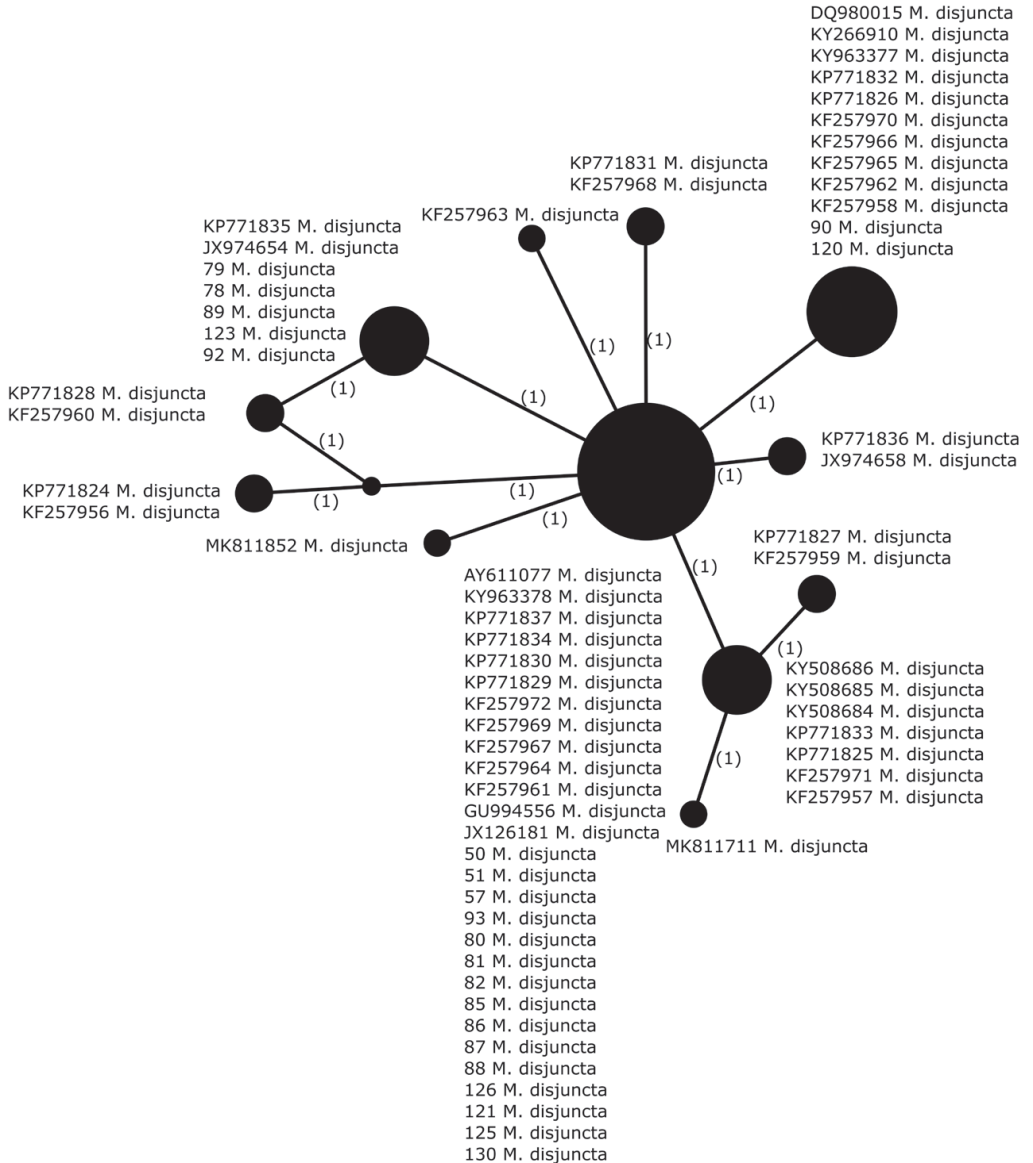
Haplotype network, based on ITS rDNA sequences from specimens of *Montaneliadisjuncta*. Newly-generated sequences are described with isolate numbers preceding the species names. Sequences downloaded from GenBank are described with their accession numbers. Mutational changes are presented as numbers in brackets near lines between haplotypes.

**Figure 7.**
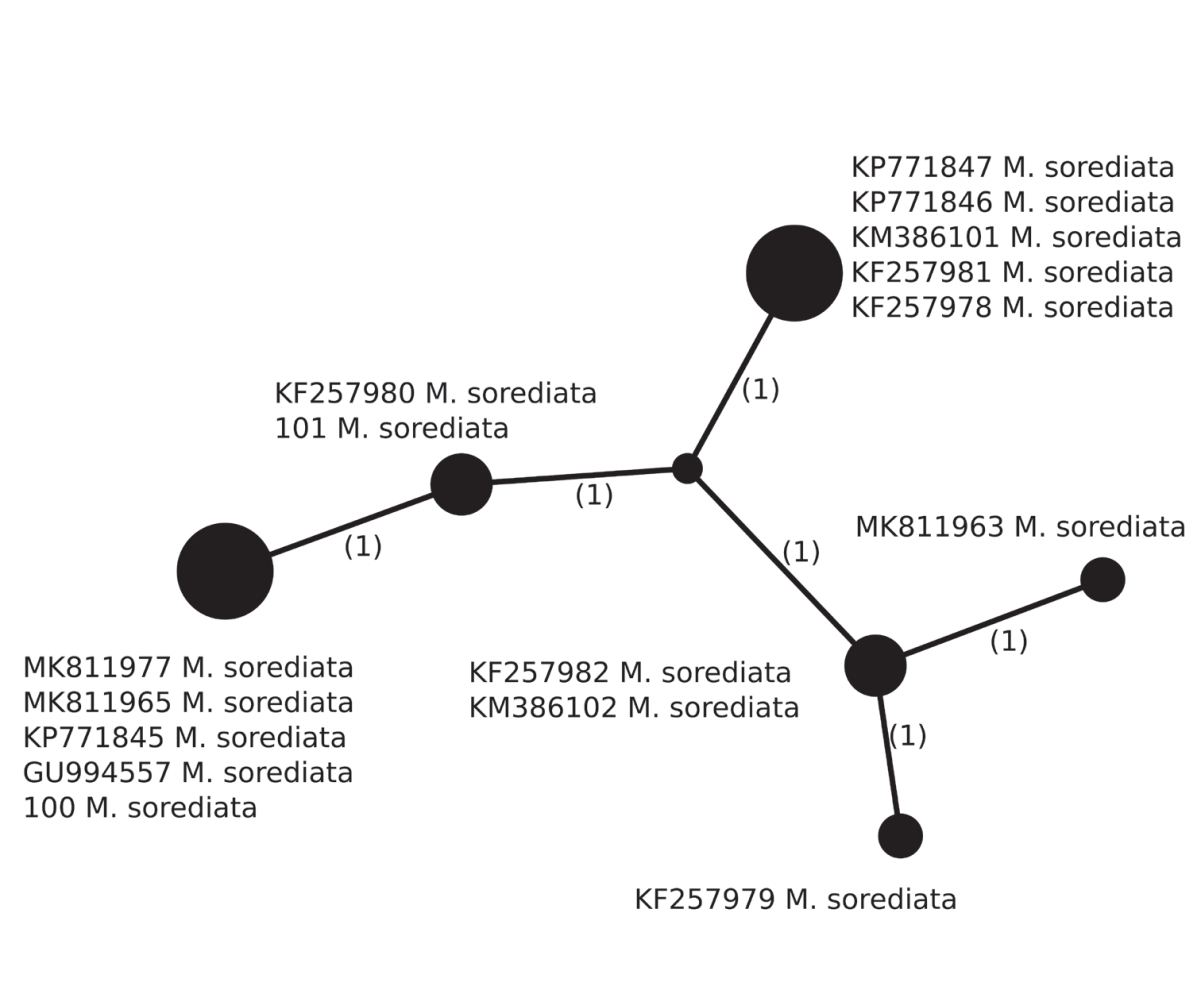
Haplotype network, based on ITS rDNA sequences from specimens of *Montaneliasorediata*. Newly-generated sequences are described with isolate numbers preceding the species names. Sequences downloaded from GenBank are described with their accession numbers. Mutational changes are presented as numbers in brackets near lines between haplotypes.

We constructed haplotype networks (Figs 2–7) to assess genetic variability within ITS rDNA marker for each species, including newly-collected specimens and data were downloaded from GenBank. The number of haplotypes found for each taxon ranged from five (in *Melaneliastygia*) to 12 (in *Melaneliahepatizon* and *Montaneliadisjuncta*); however, their numbers seem to be correlated with the abundance of specimens tested, which ranged from 10 (in *Melaneliaagnata*) to 67 (in *Montaneliadisjuncta*). Moreover, we also calculated nucleotide diversity for each dataset and found lower values for *Montaneliadisjuncta* and *Cetrariacommixta* (0.00380 and 0.00405, respectively) and higher values for *Melaneliaagnata*, *M.hepatizon* and *M.stygia* (0.01552, 0.01421 and 0.01418, respectively) (Table 2).

**Table 2.** List of haplotypes identified in this study and their geographical distribution. Nucleotide diversity for each species is also presented, and the newly generated sequences are in bold.

Haplotypes number	North America	North Europe	Central Europe	West Europe	Asia	Nucleotide diversity
***Cetrariacommixta***						
1	KF257934 Greenland	AF451796 Finland	**37 Poland**			
	KF257937 Greenland	KY266843 Norway	**97 Poland**			0.00405
	KF257938 Greenland	KC990132 Sweden	**129 Germany**			
		GU994554 Sweden				
2			**36 Poland**			
			**124 Poland**			
3	AF451797 Canada					
4	KF257939 Greenland					
5	KF257936 Greenland					
6	KF257935 Greenland					
7				GU994555 Spain		
***Melaneliaagnata***						
1		KY508672 Iceland				
		KY508673 Iceland				0.01552
		KY963373 Iceland				
		KY963374 Iceland				
2			**102 Poland**			
			**103 Poland**			
3	KF257940 Greenland					
4	KF257941 Greenland					
5	KF257942 Greenland					
6		MK257942 Norway				
***Melaneliahepatizon***						
1	KF257943 Greenland	KY508678 Iceland	**98 Poland**			
	KF257944 Greenland	KY508680 Iceland	**111 Poland**			0.01421
		KY508679 Norway	**128 Germany**			
		MK812188 Norway				
2	KF257945 Greenland	KY508675 Iceland	**109 Poland**			
	KF257946 Greenland	KY508676 Iceland				
	KF257947 Greenland					
	KF257949 Greenland					
	KF257951 Greenland					
3		KY508674 Iceland	**95 Poland**			
		KY508677 Iceland	**110 Poland**			
		KY266879 Iceland				
		KY266879 Norway				
		DQ980016 Sweden				
4			**83 Poland**	AF451776 Italy		
			**96 Poland**			
			**113 Poland**			
			**116 Slovakia**			
			**119 Poland**			
			**122 Poland**			
5	KF257950 Greenland					
	KF257953 Greenland					
6	KF257952 Greenland					
7	KF257948 Greenland					
8	AF141369 Canada					
9		KY963376 Iceland				
10		MK812512 Norway				
11			**91 Poland**			
12			**117 Slovakia**			
***Melaneliastygia***						
1		AY611097 Finland	AY611121 Austria	AF451775 Italy		
		MK812608 Norway	**40 Poland**			0.01418
			**42 Austria**			
			**94 Poland**			
			**104 Poland**			
			**108 Poland**			
			**112 Poland**			
			**127 Czech Republic**			
2		KY508681 Island				
		KY508682 Island				
		KY508683 Island				
		KY963375 Island				
3	KF257954 Greenland					
	KF257955 Greenland					
4		AF115763 Finland				
5		MK812312 Norway				
***Montaneliadisjuncta***						
1	KF257964 Canada	KY963378 Iceland	AY611077 Austria		GU994556 India	
	KF257967 Canada	KF257961 Norway	**50 Poland**		KF257972 India	0.00380
	KF257969 Canada	KP771829 Norway	**51 Poland**		KP771837 India	
	KP771830 Canada	KP771834 Sweden	**57 Poland**			
	JX126181 USA		**80 Poland**			
			**81 Poland**			
			**82 Poland**			
			**85 Poland**			
			**86 Poland**			
			**87 Poland**			
			**88 Poland**			
			**93 Poland**			
			**121 Poland**			
			**125 Poland**			
			**126 Poland**			
			**130 Czech Republic**			
2	KF257962 Canada	KY963377 Iceland	**90 Poland**			
	KF257965 Canada	KY266910 Norway	**120 Poland**			
	KF257966 Canada	DQ980015 Sweden				
	KP771832 Greenland					
	KF257958 Greenland					
	KF257970 Greenland					
	KP771826 Greenland					
3	KF257957 Greenland	KY508684 Iceland				
	KF257971 Greenland	KY508685 Iceland				
	KP771825 Greenland	KY508686 Iceland				
	KP771833 Greenland					
4	-		**78 Poland**			
		JX974654 United Kingdom	**79 Poland**			
		KP771835 United Kingdom	**89 Poland**			
			**92 Poland**			
			**123 Poland**			
5	KF257956 Canada					
	KP771824 Canada					
6	JX974658 Canada					
	KP771836 Canada					
7	KF257963 Canada					
8	KF257959 Greenland					
	KP771827 Greenland					
9	KF257968 USA					
	KP771831 USA					
10	KF257960 USA					
	KP771828 USA					
11		MK811852 Norway				
12		MK811711 Norway				
***Montaneliasorediata***						
1		MK811977 Norway	**100 Poland**			
		MK811965 Norway				0.00830
		GU994557 Sweden				
		KP771845 Sweden				
2	KF257978 USA				KF257981 Russia	
	KP771846 USA				KP771847 Russia	
					KM386101 Japan	
3	KF257980 USA		**101 Poland**			
4	KM386102 Canada					
	KF257982 Canada					
5	KF257979 Canada					
6		MK811963 Norway				

### ﻿Characteristics of the studied species

#### ﻿ Cetraria commixta

Taxon classificationFungiLecanoralesParmeliaceae

(Nyl.) Th. Fr.

Lichenographia Scandinavica 1:109 (1871) ≡ Platysmacommixtum Nyl., Synopsis methodica lichenum 1:310 (1860) ≡ Melaneliacommixta (Nyl.) A. Thell, Nova Hedwigia 60:417 (1995) ≡ Cetrariellacommixta (Nyl.) A. Thell & Kärnefelt, Mycological Progress 3:309 (2004).

##### Description.

*C.commixta* is a foliose species with elongated, smooth and flat lobes, 0.25–2.5 mm broad, which are thick on the margins and rounded at the ends (Szczepańska and Kossowska 2017). Its upper surface is glossy, olive-brown to dark brown or almost black. The lower surface is pale brown, but darker in the centre, with single, dark rhizines. *C.commixta* possess rounded or slightly elongated pseudocyphellae, which are present only on the margins and edges of lobes and cylindrical, marginal pycnidia, producing hyaline, citriform conidia (3–4 × 1–1.5 µm). Apothecia are marginal, constricted at base, 0.2–7 mm diam., with hyaline, ellipsoid to oblong-ellipsoid ascospores (6–8 × 4–6 μm).

##### Chemistry.

α-collatolic acid (chemotype I) or no substances (chemotype III).

##### Distribution.

*C.commixta* is a circumpolar and arctic-alpine species (Otte et al. 2005), growing mainly in mountain sites, in open places with high precipitation, on natural acid, siliceous rocks in North America and Europe. Available molecular data concern samples collected in North America (Canada, Greenland), as well as North (Finland, Norway, Sweden) and West (Spain) Europe.

##### Haplotypes differentiation.

We identified seven different haplotypes (Fig. 2, Table 2) within *C.commixta* (n = 17) that differ from each other in one or two positions, except for a single Canadian sample that differs in at least eight positions. The most common haplotype was found in ten specimens occurring in Greenland and North and Central Europe, amongst them being three newly-sequenced specimens (samples 37 and 97 from Poland and sample 129 from Germany). Moreover, two Polish specimens (samples 36 and 124 from the Sudety Mountains) represent a unique haplotype that differs from the most common one in a single position. Five haplotypes identified in our dataset were represented by single specimens originating from Greenland (3 haplotypes), Canada or Spain.

#### ﻿ Melanelia agnata

Taxon classificationFungiLecanoralesParmeliaceae

(Nyl.) A. Thell

Nova Hedwigia 60:416 (1995) ≡ Platysmaagnatum Nyl., Flora, Jena 60:562 (1877) ≡ Cetrariaagnata (Nyl.) Kristinsson, Lichenologist 6:144 (1974).

##### Description.

*M.agnata* has foliose thallus with flat, smooth, 0.25–2 mm broad lobes which are thicker on the margins and rounded at the ends (Szczepańska and Kossowska 2017). The upper surface is glossy, olive-brown to dark brown. The lower surface is pale brown to dark brown in the centre, with single, dark rhizines. *M.agnata* possess pseudocyphellae which are larger on the lobe margins and smaller, punctiform on the upper surface of the lobes. Pycnidia are mainly marginal to laminal, partially immersed and globose with hyaline bacilliform conidia (4.5–5.5 × 1 µm). Apothecia are not seen in examined material.

##### Chemistry.

No secondary metabolites were detected by TLC.

##### Distribution.

*M.agnata* is a rare taxon occurring in arctic and boreal regions in North America and Europe, growing in open stands on siliceous and basalt rocks (Otte et al. 2005). Available molecular data concern samples collected only in North America (Greenland) and North Europe (Iceland, Norway).

##### Haplotypes differentiation.

Six different haplotypes were identified in *M.agnata* (n = 10), of which two Polish specimens, collected in the Karpaty Mountains, have the same, not previously known, haplotype (Fig. 3, Table 2). It differs from other haplotypes in at least seven positions. However, the remaining specimens originate from Greenland, Iceland or Norway and no other samples from Central Europe have been sequenced until now. Four Icelandic specimens have the same haplotype, which is similar to the haplotype from Norwegian specimens. In contrast, Icelandic haplotypes differ from Greenlandic haplotypes in at least eight positions. Whether their genetic diversity supports conclusions from previous papers suggesting potentially unrecognised species lineages in the *M.agnata* genus (Leavitt et al. 2014; Xu et al. 2017) remains unresolved and should be further studied.

#### ﻿ Melanelia hepatizon

Taxon classificationFungiLecanoralesParmeliaceae

(Ach.) A. Thell

Nova Hedwigia 60:419 (1995) ≡ Lichenhepatizon Ach., Lichenographiae Sueciae Prodromus 110 (1798) ≡ Cetrariahepatizon (Ach.) Vain., Termeszetrajzi Füzetek 22:278 (1899).

##### Description.

*M.hepatizon* is foliose species with flat lobes that are 0.25–2.5 mm broad and thick at the margins (Szczepańska and Kossowska 2017). Its upper surface is glossy, brown to almost black. The lower surface is dark brown to black, paler near the margins, with single, dark rhizines. Pseudocyphellae are mainly present on the margins and edges of lobes. Pycnidia are marginal, but sometimes also laminal, sessile, globose to stalked, slightly elongated or cylindrical with hyaline, bacilliform conidia (3–5 × 1 µm). Apothecia are marginal to laminal, sessile, with hyaline, ellipsoid to oblong-ellipsoid ascospores (6–8 × 4–6 μm).

##### Chemistry.

Stictic and norstictic acids.

##### Distribution.

*M.hepatizon* is a circumpolar and arctic-alpine species occurring from oceanic to continental sites on siliceous rocks in North America and Europe (Otte et al. 2005). Available molecular data concern samples collected in North America (Canada, Greenland) as well as North (Iceland, Norway, Sweden) and West (Italy) Europe.

##### Haplotypes differentiation.

A higher number of haplotypes was detected in *M.hepatizon* (n = 40), in which we identified 12 haplotypes (Fig. 4, Table 2). Amongst newly-sequenced specimens, we identified six haplotypes. Some are more common and were previously found in Greenland, Iceland, Italy, Norway or Sweden. In contrast, others were only found in newly-sequenced specimens, such as sample 91 from the Sudety Mountains in Poland and sample 117 from the Karpaty Mountains in Slovakia. However, no geographic pattern was found in the dataset.

#### ﻿ Melanelia stygia

Taxon classificationFungiLecanoralesParmeliaceae

(L.) Essl.

Mycotaxon 7:47 (1978) ≡ Lichenstygius L., Species Plantarum 2:1143 (1753).

##### Description.

*M.stygia* has foliose thallus, composed of 0.25–1.5 mm broad, smooth and usually distinctly convex lobes (Szczepańska and Kossowska 2017). The upper surface is glossy, dark brown to almost black. The lower surface is dark brown to black, paler near the margins, with single, dark rhizines. Pseudocyphellae in this species are numerous, rounded or slightly elongated and laminal – clearly visible on the upper surface of the lobes. Pycnidia are also common, globose, laminal and immersed with hyaline, bacilliform conidia (3.5–5 × 1 µm). Apothecia are laminal, constricted at the base and 0.5–6 mm in diameter. Ascospores are hyaline, ellipsoid to oblong-ellipsoid, 6–8 × 4–6 μm in size.

##### Chemistry.

Protocetraric and fumarprotocetraric acids (Race 1) or no substances detected (Race 6).

##### Distribution.

*M.stygia* is a circumpolar and arctic-alpine species occurring mainly on siliceous rocks in upper mountain areas in North America and Europe (Otte et al. 2005). Available molecular data concern only a few samples collected in North America (Greenland) and North (Iceland, Finland, Norway) and West (Italy) Europe.

##### Haplotypes differentiation.

Amongst five identified haplotypes in *Melaneliastygia* (n = 19), all newly-sequenced specimens (five from Poland, one from Austria and one from the Czech Republic) have the same haplotype, previously reported from Austria, Finland, Italy and Norway (Fig. 5, Table 2). It differs from the haplotype identified in another Finnish specimen in two positions. Two Greenlandic specimens have the same haplotype that differs from the most common one in five positions. Four Icelandic samples have an identical haplotype that differs from the Norwegian sample in five positions; however, these samples differ in at least 13 positions from other haplotypes of *M.stygia*. Moreover, these Icelandic and one Norwegian samples form a separate clade shown in Fig. 1, in contrast to the remaining specimens of *M.stygia*. These molecular data suggest that these lineages may represent phenotypically indistinguishable cryptic species.

#### ﻿ Montanelia disjuncta

Taxon classificationFungiLecanoralesParmeliaceae

(Erichsen) Divakar, A. Crespo, Wedin & Essl.

American Journal of Botany 99:2022 (2012) ≡ Parmeliadisjuncta Erichsen, Annales Mycologici 37:78 (1939) ≡ Melaneliadisjuncta (Erichsen) Essl., Mycotaxon 7:46 (1978).

##### Description.

*M.disjuncta* possess foliose thallus composed of 0.6–1.2 mm broad, flat to slightly convex and glossy lobes (Szczepańska et al. 2015). Its upper surface is smooth, olive-brown to dark brown. Pseudocyphellae are small, rather indistinct and submarginal. Its characteristic feature is the presence of the soralia (0.2–0.5 mm in diameter), which are punctiform, irregular, usually capitate and arise on the surface or at the margins of the lobes. Soredia are granular to isidioid, dark, but appearing white when abraded. Pycnidia are rare, conidia are 6–7 × 1 μm. Apothecia are not seen in the examined material.

##### Chemistry.

Perlatolic and stenosporic acids.

##### Distribution.

*M.disjuncta* is a circumpolar species growing mainly on siliceous rocks. The geographical range of this species consists of both continental and oceanic areas of Europe and North America (Esslinger 1977; Otte et al. 2005; Hansen 2013). Available molecular data concern samples collected in North America (Canada, Greenland, USA), North (Iceland, Norway, Sweden, United Kingdom) and Central (Austria) Europe, as well as Asia (India).

##### Haplotypes differentiation.

Twelve different haplotypes were identified in *M.disjuncta* (n = 67), of which the most common haplotype occurs in Europe, North America and Asia (Fig. 6, Table 2). The highest diversity was observed in North America (Canada, Greenland, USA), for which a total of nine different haplotypes were found, including six that were exclusive for this region. We identified three different haplotypes amongst the newly-collected samples (n = 22). The most common one also occurs in other European countries, Asia and North America. The second most common also occurs in Northern Europe and North America, while the third haplotype was previously identified in specimens collected in the United Kingdom. Moreover, four different haplotypes were identified amongst specimens collected in Norway, while five haplotypes were identified in Canadian samples, of which three are unique to Canada. Three haplotypes were identified in samples from both Iceland and Greenland, two of which are common for these areas and one haplotype is unique to Greenland. Some haplotypes are represented by more than one sample originating from particular areas, such as Alaska and Maine (USA), the Yukon Territory (Canada) or Greenland. The haplotypes identified in our dataset originated from different geographical areas and two of the most common haplotypes are widely distributed in the Northern Hemisphere. Based on the presented sampling, we could not indicate any geographical pattern, neither locally nor worldwide.

#### ﻿ Montanelia sorediata

Taxon classificationFungiLecanoralesParmeliaceae

(Ach.) Divakar, A. Crespo, Wedin & Essl.

American Journal of Botany 99:2023 (2012) ≡ Parmeliastygiavar.sorediata Ach., Lichenographia Universalis 471 (1810) ≡ Melaneliasorediosa (Almb) Essl., Mycotaxon 7:47 (1978) ≡ Melaneliasorediata (Ach.) Goward & Ahti, Mycotaxon 28:94 (1987).

##### Description.

*M.sorediata* is a foliose species. Its lobes are flat to slightly convex, 0.2–0.6 mm broad, distinctly rugged and pitted at the ends (Szczepańska et al. 2017). The upper surface is smooth, dull, olive brown to dark brown. Characteristic soralia arise on the ends of the main lobes or on the smaller, erect side lobes. They are usually distinctly convex and capitate with granular to isidioid, dark soredia. Pseudocyphellae and pycnidia are absent. Apothecia are not seen in the examined material.

##### Chemistry.

Perlatolic and stenosporic acids.

##### Distribution.

*M.sorediata* is a probably circumpolar species that prefers siliceous substrates, usually in open and well-lit places. The species is mentioned as occurring in North America and Europe (Esslinger 1977; Otte et al. 2005). Available molecular data concern only a few samples collected in North America (Canada, USA), North Europe (Norway, Sweden) and Asia (India).

##### Haplotypes differentiation.

Six different haplotypes were identified in *M.sorediata* (n = 16), of which two Polish specimens, collected in the Karpaty Mountains, have two different haplotypes that differ in a single position (Fig. 7, Table 2). Interestingly, sample 101 has the same haplotype as the specimen collected in Alaska (KF257980), while sample 100 has the same haplotype as four Scandinavian specimens collected in Norway and Sweden. Another of the most common haplotypes is represented by specimens collected in Japan, Russia and the USA. Therefore, no specific geographic pattern was observed in the dataset.

## ﻿Discussion

Although several studies focused on the phylogeny of brown *Parmeliae*, in the analysed datasets, there was an evident lack of molecular data concerning this group from Central Europe. The available data included only North America (mainly Greenland), Northern Europe (Scandinavian countries) and single sequences from specimens collected in Western Europe (Spain, Italy) and Asia (India, Russia). Having the opportunity to collect data from Poland, we focused on taxa occurring in this country, such as *Cetrariacommixta*, *Melaneliaagnata*, *M.hepatizon*, *M.stygia*, *Montaneliadisjuncta* and *M.sorediata*. Additionally, in analyses, we also included newly-generated sequences from samples collected in Austria, Czech Republic, Germany and Slovakia. By supplementing the dataset with new sequences from a previously-unexplored area, we wanted to study the intraspecific internal transcribed spacer (ITS) rDNA variability of mentioned species and analyse distribution patterns of individual haplotypes. Previously, Leavitt et al. (2014) reported mean genetic distance (given as the number of nucleotide substitutions per site) in brown *Parmeliae* and found higher values in the case of *Melaneliaagnata* and *M.hepatizon* (0.013) in contrast to *Cetrariacommixta* and *M.stygia* (0.002 and 0.007, respectively). In this study, we found the highest nucleotide diversity in *Melaneliaagnata* and *M.hepatizon* (0.01552 and 0.01421, respectively), but also in *M.stygia* (0.01418) as a result of additional sampling.

In our study, the haplotype networks illustrated that single-locus haplotypes and clades have no geographic clustering and cannot be useful in defining the species boundaries within brown *Parmeliae*. Haplotypes are dispersed amongst the sites and clades do not show apparent association with spatial location, as reported in literature data (Werth and Sork 2008; Starosta and Svoboda 2020). In addition, many of the analysed haplotypes of brown *Parmeliae* are widely distributed and, in many cases, the same haplotypes are shared between temperate and polar populations. What is more, all taxa, except *Melaneliastygia*, seem to be monophyletic and newly-sequenced specimens cluster together with other representatives of the species downloaded from GenBank. The extremely wide geographical distribution of mycobiont haplotypes has been observed in some other species, such as *Cavernulariahultenii* (Printzen et al. 2003), *Cetrariaaculeata* (Fernández-Mendoza et al. 2011) and *Cladoniasubcervicornis* (Printzen and Ekman 2003). In the first two cases, this phenomenon is assigned to lichens characterised by vegetative propagation and interpreted as evidence for ancestral polymorphisms and slow genetic drift (Printzen et al. 2003). This finding conforms well with the results of our study on Parmeliaceae, which are usually sterile species, reproducing by soredia (*Montanelia*) and conidia (*Cetraria*, *Melanelia*).

Although representatives of brown *Parmeliae* are known from both Hemispheres (Otte et al. 2005), all species studied in this paper represent circumpolar distribution and occur only on northern continents. The specimens used for the analyses originated mainly from mountain areas of Poland, both the Carpathians and the Sudetes; however, the range of sampling seems to be representative for this part of Europe. The number of analysed haplotypes representing different geographical regions was comparable for each taxon; nevertheless, the number of *Melaneliaagnata* and *Montaneliasorediata* samples remain very small. Due to the newly-generated molecular data covering Central Europe, we were able to compare the haplotype distribution in this area with other parts of the world. Unfortunately, the data available for discussed lichens taxa include, almost exclusively, specimens from North America and Northern Europe; the data concerning Asia and Southern Europe are not sufficient to make a reliable comparison possible. In almost all analysed taxa, stronger genetic differentiation was found amongst North American populations, with a few haplotypes unique for this part of the world, especially for Greenland. Specimens occurring in Central Europe have lower haplotype diversification and many of these haplotypes have wide geographical distribution (Table 2). Nevertheless, it seems that the number of analysed sequences is still insufficient to indicate high diversity areas (hotspots), species speciation centres or glacial refugia. Although the numbers of haplotypes correlated with the number of specimens tested, two species occurring in Poland (*Melaneliaagnata* and *M.stygia*) clearly indicate a very low level of genetic diversity. Both species are rare in Poland and their distribution is limited to the high mountain regions (Szczepańska and Kossowska 2017). Low genetic diversity and limited occurrence suggest considering both taxa as critically endangered in Poland.

In recent years, it has been proved that cryptic species-level lineages are very common amongst lichen-forming fungi (Crespo and Pérez-Ortega 2009; Crespo and Lumbsch 2010; Lumbsch and Leavitt 2011). At the same time, it has been shown that phenotypic variation is not always ‘sensitive’ enough for delimitation and description of new taxa. Modern methods of genetic analysis are recommended as an additional tool for this purpose (Molina et al. 2011; de Paz et al. 2012; Leavitt et al. 2013; Renner 2016). At the same time, it is necessary to include other evidence, such as chemistry, ecology, geography and morphology, for the proper delimitation of lichenised fungi species (Hawksworth 1976; Dayrat 2005; Crespo and Pérez-Ortega 2009). Such careful and versatile analysis of distinct phylogenetic lineages may lead to recognising some previously-overlooked characteristics (Kroken and Taylor 2001; del Prado et al. 2007; Frolov et al. 2016; Leavitt et al. 2016; Szczepańska et al. 2020). In the recent review paper, Lücking et al. (2021) proposed a detailed protocol for consistent taxonomy of lichen-forming fungi. The integrative taxonomy employing phylogeny, reproductive biology and phenotype should be used to delimit species (Lücking et al. 2020). Aime et al. (2021) recommended circumscription of new taxa, based on an appropriate sampling of multiple representatives from different collections for which multi-loci analyses should be performed. They also noted that description of a new species, based on single-locus phylogenetic analyses, could only be done in exceptional cases. The errors caused by contaminant sequences, laboratory mix-ups and chimeric sequences should be avoided for proper establishment of novel taxa, based on molecular data only (Lücking et al. 2021). Therefore, it is crucial to employ unlinked loci from different parts of the genome, even though the ITS rDNA marker is widely used in DNA barcoding of fungal taxa.

We analysed phenotypic diversity of samples representing individual haplotypes in our studies. However, in morphological, anatomical and chemical analyses, we observed that phenotypic characters of individuals representing different haplotypes are homogeneous and no visible distinctive features for samples with different geographic distribution were recognised. Recent molecular studies of one of the analysed genus – *Melanelia*, suggested previously unrecognised species-level diversity within this taxon (Divakar et al. 2012; Leavitt et al. 2014; Xu et al. 2017). However, the authors based their assumptions primarily on phylogenetic analyses without considering phenotypic features. Therefore, we have decided to analyse differences in morphology, anatomy and chemistry of *M.stygia* and *M.agnata* specimens originating from different geographic regions (Greenland, Iceland and Central Europe).

**Figure 8.**
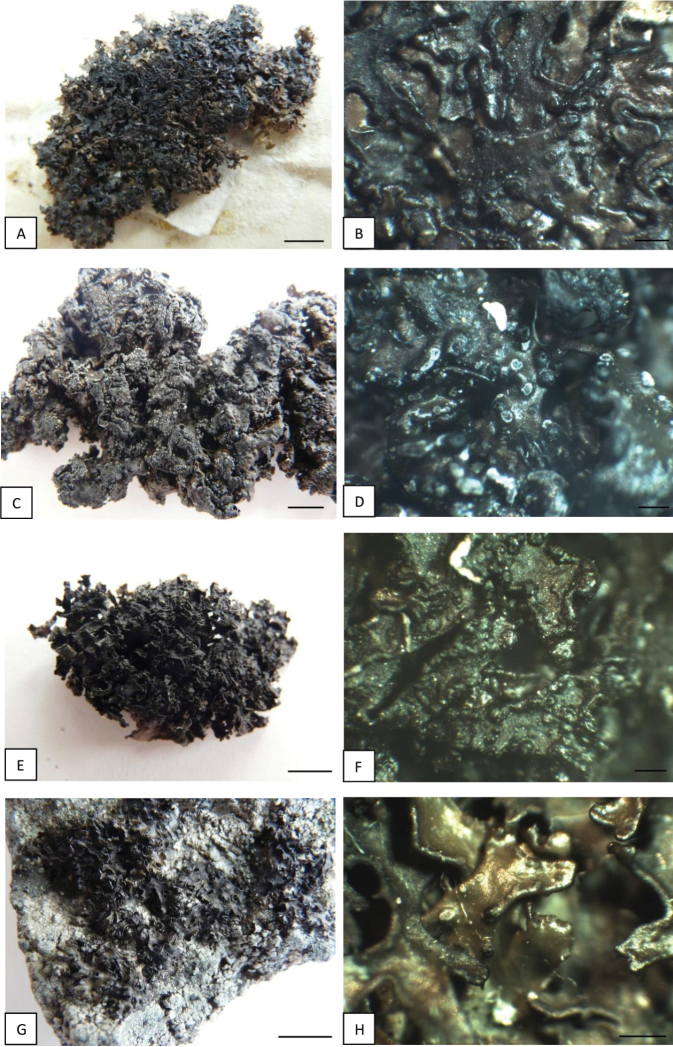
*Melaneliaagnata* specimens treated **A***Melaneliaagnata* H-NYL 36086 (holotype) **B***Melaneliaagnata*, H-NYL 36086 (holotype) **C***M.agnata*, AMNH 27562 (Iceland) **D***M.agnata*, AMNH 30974 (Iceland) **E***M.agnata*, C 19019 (Greenland) **F***M.agnata*, C 19019 (Greenland) **G***M.agnata*, Szczepańska 1050, WRSL (Poland) **H***M.agnata*, Szczepańska 1050, WRSL (Poland). Scale bars: 0.5 cm (**A, C, E, G**); 0.5 mm (**B, D, F**); 1 mm (**H**).

*Melaneliaagnata* is a rare lichen recorded in North America and some European countries, such as Austria, Iceland, Norway, Poland, Russia, Sweden, Switzerland and Slovakia (Westberg et al. 2004; Hawksworth et al. 2008; Szczepańska and Kossowska 2017). The analysed holotype of *Melaneliaagnata* is characterised by small (ca. 3.0 cm in diam.), foliose, olive-brown to dark-brown thallus, composed of flat, shiny, 0.25–2 mm broad, smooth lobes with thicker margins (Fig. 8A). Its lower surface is pale brown with single, dark rhizines. Polish (Figs 8G and H) and Greenlandic (Fig. 8E and F) specimens comply with the type. However, Icelandic material differs in a larger thallus size (up to 10 cm in diam.) and the appearance of the lobes, which are more convex than flat, 1–5 mm broad and distinctly wrinkled (Fig. 8C). Thell (1995) made an interesting taxonomic description of *M.agnata*, in which he noted that its thallus could reach up to 10 cm diam. However, in his research, Thell (1995) analysed only a few specimens, including one from Iceland (Kristinsson 14781, GZU, LD) and treated them all as a single taxon. A similar situation applies to conidia, reaching 5–7.5 µm in *M.agnata*, according to Thell (1995). Pycnidia observed in Icelandic specimens are usually marginal (Fig. 8D), very often double and produce bifusiform conidia, 4.5–6 × 1 µm, in contrast to the type specimen, which pycnidia are simple, marginal to laminal (Fig. 8B) with smaller conidia, at 3.5–5 × 1 µm. Pseudocyphellae are always whitish, rounded or irregular, marginal and laminal in all analysed material; they are much more abundant in specimens from Iceland (Fig. 8D). None of the Icelandic specimens had apothecia, so their anatomical analysis was impossible. All material was chemically homogeneous and no secondary metabolites were detected by thin-layer chromatography (TLC), which is consistent with other descriptions (Thell 1995; Xu et al. 2017).

*Melaneliastygia* is a much more common species than *M.agnata*. In Europe, it was recorded in the upper mountain areas of Austria, the Czech Republic, Germany, Great Britain, Poland, Romania, Russia, Slovakia, Switzerland and Ukraine (Hawksworth et al. 2008).

**Figure 9.**
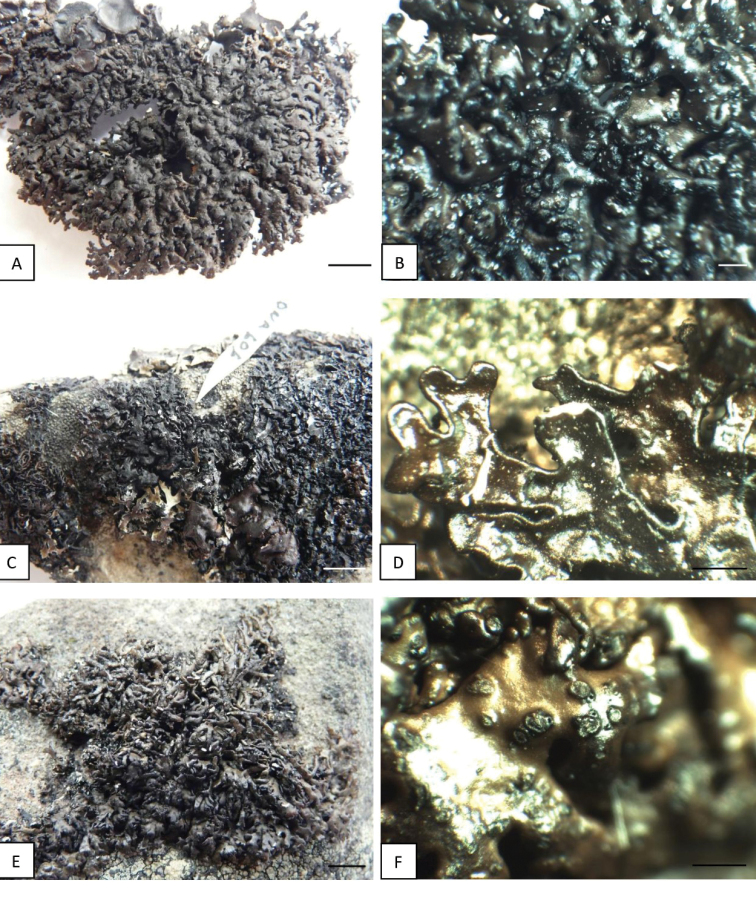
*Melaneliastygia* specimens treated **A***M.stygia*, AMNH 28243 (Iceland) **B***M.stygia*, AMNH 16894 (Iceland) **C***M.stygia*, C 19893 (Greenland) **D***M.stygia*, C 19893 (Greenland) **E***M.stygia*, Szczepańska 1160, WRSL (Poland) **F***M.stygia*, Szczepańska 737, WRSL (Austria). Scale bars: 0.5 cm (**A, C, E)**;1 mm (**B, D**); 0.5 mm (**F**).

After phenotypic studies, we have concluded that all material is homogeneous and none of the analysed morphological and anatomical features coincides with geographically-distinct *M.stygia* populations (Fig. 9A–F). However, some differences may be observed in the secondary chemistry. In his paper, Esslinger (1977) recognised six chemical races within *M.stygia*. He stated that some of them are broadly distributed and others are more frequent in particular regions. All the currently-examined samples originating from Greenland and Central Europe belong to Race 1, containing fumaroprotocetraric and protocetraric acids. Specimens from Iceland represent Race 6, without secondary metabolites. Both races are known to occur in Japan, North America and Europe; however, there is a possibility that Race 6 is the only chemical Race occurring in Iceland. Production of some secondary metabolites may be induced by environmental factors (Culberson 1986; Leavitt et al. 2011) and does not always correspond with molecular data. Moreover, chemical differences can be observed within some recognised haplotype groups and even in the same haplotype (Matteucci et al. 2017). At the same time, chemical characters may be successfully used to support delimitation of lichen taxa, but in any case, they cannot be treated as an exclusive diagnostic trait (Elix et al. 2009; Spribille et al. 2011; Leavitt et al. 2013; Onut-Brännström et al. 2018; Mark et al. 2019,).

In conclusion, we can state that all of the potential species lineages within *Melaneliaagnata* and *M.stygia* are cryptic, with very slight morphological, anatomical and chemical variation. We were unable to distinguish any distinctive feature that could be considered diagnostic and useful for the delimitation of new species, except molecular variation. The phenotypic differences mentioned above may reflect environmental or climate conditions, such as temperature, light, humidity or substrate and may not be connected with genetic differences. However, this study was limited to a small number of samples and one genetic marker, ITS; therefore, we refrain from describing new species because further study is pending. We suggest that an extended phylogeographic study is necessary and an increase in the number of herbarium specimens would probably give additional information. Even though our analyses complement the knowledge on lichens in Central Europe, many areas remain insufficiently explored. Additional sampling from Asia and Southern Europe may bring new data on the phylogenetic and phenotypic diversity of species from the brown *Parmeliae* group.

## Supplementary Material

XML Treatment for Cetraria commixta

XML Treatment for Melanelia agnata

XML Treatment for Melanelia hepatizon

XML Treatment for Melanelia stygia

XML Treatment for Montanelia disjuncta

XML Treatment for Montanelia sorediata

## Supplementary materials

Supplementary material 1 Figure S1 Data type Images. Explanation note Phylogenetic relationships of brown Parmeliae, based on Bayesian analysis of the ITS rDNA dataset. Posterior probabilities and Maximum Likelihood bootstrap values are shown near the internal branches. Newly-generated sequences are described with isolate numbers preceding the species names and are marked in bold. GenBank accession numbers of sequences downloaded from GenBank are listed on the tree with species names. https://binary.pensoft.net/file/626681This dataset is made available under the Open Database License (http://opendatacommons.org/licenses/odbl/1.0/). The Open Database License (ODbL) is a license agreement intended to allow users to freely share, modify, and use this Dataset while maintaining this same freedom for others, provided that the original source and author(s) are credited.Katarzyna Szczepańska, Beata Guzow-Krzemińska, Jacek Urbaniak

## References

[B1] Aime MC Miller AN Aoki T Bensch K Cai L Crous PW Hawksworth DL Hyde KD Kirk PM Lücking R May TW Malosso E Redhead SA Rossman AY Stadler M Thines M Yurkov AM Zhang N Schoch CL (2021) How to publish a new fungal species, or name, version 3.0. IMA Fungus 12: е11. 10.1186/s43008-021-00063-1 PMC8091500 33934723

[B2] Altschul SF Madden TL Schäffer AA Zhang J Zhang Z Miller W Lipman J (1997) Gapped BLAST and PSI-BLAST: a new generation of protein database search programs. Nucleic Acids Research 25: 3389–3402. 10.1093/nar/25.17.3389 9254694 PMC146917

[B3] Blanco O Crespo A Divakar PK Esslinger TL Hawksworth DL Lumbsch H (2004) *Melanelixia* and *Melanohalea*, two new genera segregated from *Melanelia* (Parmeliaceae) based on molecular and morphological data. Mycological Research 108: 873–884. 10.1017/S0953756204000723 15449592

[B4] Blanco O Crespo A Divakar PK Elix JA Lumbsch HT (2005) Molecular phylogeny of parmotremoid lichens (Ascomycota, Parmeliaceae). Mycologia 97: 150–159. 10.1080/15572536.2006.11832848 16389966

[B5] Clement M Snell Q Walker P Posada D Crandall K (2002) TCS: Estimating gene genealogies. Parallel and Distributed Processing Symposium, International Proceedings 2: е184. 10.1109/IPDPS.2002.1016585

[B6] Crespo A Pérez-Ortega S (2009) Cryptic species and species pairs in lichens: a discussion on the relationship between molecular phylogenies and morphological characters. Anales del Jardín Botánico de Madrid 66: 71–81. 10.3989/ajbm.2225.

[B7] Crespo A Lumbsch HT (2010) Cryptic species in lichen-forming fungi. IMA fungus 1: 167–170. 10.5598/imafungus.2010.01.02.09 22679576 PMC3348775

[B8] Crespo A Divakar PK Hawksworth DL (2011) Generic concepts in parmelioid lichens, and the phylogenetic value of characters used in their circumscription. Lichenologist (London, England) 43: 511–535. 10.1017/S0024282911000570

[B9] Crespo A Kauff F Divakar PK del Prado R Pérez-Ortega S Amo de Paz G et al. (2010) Phylogenetic generic classification of parmelioid lichens (Parmeliaceae, Ascomycota) based on molecular, morphological and chemical evidence. Taxon 59: 1735–1753. 10.1002/tax.596008

[B10] Culberson CF (1972) Improved conditions and new data for identification of lichen products by standardized thin-layer chromatographic method. Journal of Chromatography A 72: 113–125. 10.1016/0021-9673(72)80013-X. 5072880

[B11] Culberson WL (1986) Chemistry and sibling speciation in the lichen-forming fungi: ecological and biological considerations. Bryologist 89: 123–131. 10.2307/3242752

[B12] Darriba D Taboada GL Doallo R Posada D (2012) jModelTest 2: more models, new heuristics and parallel computing. Nature Methods 9: е772. 10.1038/nmeth.2109 PMC4594756 22847109

[B13] Dayrat B (2005) Towards integrative taxonomy. Biological Journal of the Linnean Society 85: 407–417. 10.1111/j.1095-8312.2005.00503.x

[B14] de Paz GA Cubas P Crespo A Elix JA Lumbsch HT (2012) Transoceanic dispersal and subsequent diversification on separate continents shaped diversity of the *Xanthoparmeliapulla* group (Ascomycota). PLоS ONE 7(6): e39683. 10.1371/journal.pone.0039683 PMC3379998 22745810

[B15] del Prado R Ferencová Z Armas-Crespo V de Paz GA Cubas P Crespo A (2007) The arachiform vacuolar body: an overlooked shared character in the ascospores of a large monophyletic group within Parmeliaceae (*Xanthoparmelia* clade, Lecanorales). Mycological Research 111: 685–692. 10.1016/j.mycres.2007.04.002 17601715

[B16] Divakar PK Del-Prado R Lumbsch HT Wedin M Esslinger TL Leavitt SD Crespo A (2012) Diversification of the newly recognized lichen-forming fungal lineage *Montanelia* (Parmeliaceae, Ascomycota) and its relation to key geological and climatic. American Journal of Botany 99: 2014–2026. 10.3732/ajb.1200258 23204485

[B17] Domaschke S Fernandez-Mendoza FA García M Martín M Printzen C (2012) Low genetic diversity in Antarctic populations of the lichen-forming ascomycete *Cetrariaaculeata* and its photobiont. Polar Research 31(1): е17353. 10.3402/polar.v31i0.17353

[B18] Doyle JJ Doyle JL (1987) A rapid DNA isolation procedure for small quantities of fresh leaf tissue. Phytochemical Bulletin 19: 11–15.

[B19] Elix JA Corush J Lumbsch HT (2009) Triterpene chemosyndromes and subtle morphological characters characterise lineages in the *Physciaaipolia* group in Australia (Ascomycota). Systematics and Biodiversity 7: 479–487 10.1017/S1477200009990223.

[B20] Esslinger TL (1977) A chemosystematic revision of the brown Parmeliae. Journal of the Hattori Botanical Laboratory 42: 1–211.

[B21] Esslinger TL (1978) A new status for the brown Parmeliae. Mycotaxon 7: 45–54.

[B22] Fernández-Mendoza F Domaschke S García MA Jordan P Martín MP Printzen C (2011) Population structure of mycobionts and photobionts of the widespread lichen *Cetrariaaculeata*. Molecular Ecology 20: 1208–1232. 10.1111/j.1365-294X.2010.04993.x 21324011

[B23] Frolov I Vondrá J Fernández-Mendoza F Wilk K Khodosovtsev A Halıcı MG (2016) Three new, seemingly-cryptic species in the lichen genus *Caloplaca* (Teloschistaceae) distinguished in two-phase phenotype evaluation. In Annales Botanici Fennici 53: 243–262. 10.5735/085.053.0413

[B24] Galtier N Gouy M Gautier C (1996) SEAVIEW and PHYLO_WIN: two graphic tools for sequence alignment and molecular phylogeny. Computational Applied Biosciences 12: 543–548. 10.1093/bioinformatics/12.6.543 9021275

[B25] Gouy M Guindon S Gascuel O (2010) SeaView version 4: a multiplatform graphical user interface for sequence alignment and phylogenetic tree building. Molecular Biology and Evolution 27: 221–224. 10.1093/molbev/msp259 19854763

[B26] Guzow-Krzemińska B Węgrzyn G (2003) A preliminary study on the phylogeny of the genus *Melanelia* using nuclear large subunit ribosomal DNA sequences. Lichenologist 35: 83–86. 10.1006/lich.2002.0429

[B27] Hansen ES (2013) Lichens from three localities in Central West Greenland with notes on their climatic preferences. Botanica Lithuanica 19: 28–36. 10.2478/botlit-2013-0004

[B28] Hawksworth DL (1976) Lichen chemotaxonomy. In: Brown DH Hawksworth DL Bailey RH (Eds) Lichenology: progress and problems.Academic Press, London, 139–184.

[B29] Hawksworth DL Blanco O Divakar PK Ahti T Crespo A (2008) A first checklist of parmelioid and similar lichens in Europe and some adjacent territories, adopting revised generic circumscriptions and with indications of species distributions. Lichenologist 40: 1–21. 10.1017/S0024282908007329

[B30] Huelsenbeck JP Ronquist F (2001) MrBayes: Bayesian inference of phylogenetic trees. Bioinformatics 17: 754–755. 10.1093/bioinformatics/17.8.754 11524383

[B31] Katoh K Misawa K Kuma K Miyata T (2002) MAFFT: a novel method for rapid multiple sequence alignment based on fast Fourier transform. Nucleic Acids Research 30: 3059–3066. 10.1093/nar/gkf436 12136088 PMC135756

[B32] Kroken S Taylor JW (2001) A gene genealogical approach to recognize phylogenetic species boundaries in the lichenized fungus *Letharia*. Mycologia 93: 38–53. 10.1080/00275514.2001.12061278

[B33] Landan G Graur D (2008) Local reliability measures from sets of co-optimal multiple sequence alignments. Pacific Symposium on Biocomputing 13: 15–24. 18229673

[B34] Lanfear R Frandsen PB Wright AM Senfeld T Calcott B (2016) PartitionFinder 2: new methods for selecting partitioned models of evolution for molecular and morphological phylogenetic analyses. Molecular Biology and Evolution 34: 772–773. 10.1093/molbev/msw260 28013191

[B35] Leavitt SD Divakar PK Ohmura Y Wang L-S Esslinger TL Lumbsch HT (2015) Who’s getting around? Assessing species diversity and phylogeography in the widely distributed lichen-forming fungal genus *Montanelia* (Parmeliaceae, Ascomycota). Molecular Phylogenetics and Evolution 90: 85–96. 10.1016/j.ympev.2015.04.029 25987532

[B36] Leavitt SD Fankhauser JD Leavitt DH Porter LD Johnson LA Clair LLS (2011) Complex patterns of speciation in cosmopolitan “rock posy” lichens-Discovering and delimiting cryptic fungal species in the lichen-forming *Rhizoplacamelanophthalma* species-complex (Lecanoraceae, Ascomycota). Molecular Phylogenetics and Evolution 59: 587–602. 10.1016/j.ympev.2011.03.020 21443956

[B37] Leavitt S Fernández-Mendoza F Pérez-Ortega S Sohrabi M Divakar P Lumbsch T Clair LLS (2013) DNA barcode identification of lichen-forming fungal species in the *Rhizoplacamelanophthalma* species-complex (Lecanorales, Lecanoraceae), including five new species. MycoKeys 7: 1–22. 10.3897/mycokeys.7.4508.

[B38] Leavitt SD Esslinger TL Hansen ES Divakar PK Crespo A Loomis BF Lumbsch HT (2014) DNA barcoding of brown Parmeliae (Parmeliaceae) species: a molecular approach for accurate specimen identification, emphasizing species in Greenland. Organisms Diversity & Evolution 14: 11–20. 10.1007/s13127-013-0147-1

[B39] Leavitt SD Esslinger TL Divakar PK Crespo A Lumbsch HT (2016) Hidden diversity before our eyes: Delimiting and describing cryptic lichen-forming fungal species in camouflage lichens (Parmeliaceae, Ascomycota). Fungal Biology 120: 1374–1391. 10.1016/j.funbio.2016.06.001 27742095

[B40] Lindblom L Ekman S (2006) Genetic variation and population differentiation in the lichen-forming ascomycete *Xanthoriaparietina* on the island Storfosna, central Norway. Molecular Ecology 15: 1545–1559. 10.1111/j.1365-294X.2006.02880.x 16629810

[B41] Lumbsch HT Leavitt SD (2011) Goodbye morphology? A paradigm shift in the delimitation of species in lichenized fungi. Fungal Diversity 50: 59–72. 10.1007/s13225-011-0123-z

[B42] Lücking R Aime MC Robberts B Miller AN Ariyawansa HA Aoki T Cardinali G Crous PW Druzhinina IS Geiser DM Hawksworth DL Hyde KD Irinyi L Jeewon R Johnston PR Kirk PM Malosso E May TW Meyer W Öpik M Robert V Stadler M Thines M Vu D Yurkov AM Zhang N Schoch CL (2020) Unambiguous identification of fungi: where do we stand and how accurate and precise is fungal barcoding? IMA Fungus 11: е14. 10.1186/s43008-020-00033-z PMC7353689 32714773

[B43] Lücking R Leavitt SD Hawksworth DL (2021) Species in lichen-forming fungi: balancing between conceptual and practical considerations, and between phenotype and phylogenomics. Fungal Diversity. 10.1007/s13225-021-00477-7

[B44] Mark K Randlane T Thor G Hur JS Obermayer W Saag A (2019) Lichen chemistry is concordant with multilocus gene genealogy in the genus *Cetrelia* (Parmeliaceae, Ascomycota). Fungal Biology 123: 125–139. 10.1016/j.funbio.2018.11.013 30709518

[B45] Matteucci E Occhipinti A Piervittori R Maffei ME Favero-Longo SE (2017) Morphological, secondary metabolite and ITS (rDNA) variability within usnic acid-containing lichen thalli of *Xanthoparmelia* explored at the local scale of rock outcrop in W-Alps. Chemistry & Biodiversity 14: e1600483. 10.1002/cbdv.201600483 28296214

[B46] Miller MA Pfeiffer W Schwartz T (2010) Creating the CIPRES Science Gateway for inference of large phylogenetic trees. In: Proceedings of the Gateway Computing Environments Workshop (GCE), 14 November 2010, New Orleans, 1–8. 10.1109/GCE.2010.5676129

[B47] Molina M Del-Prado R Divakar PK Sánchez-Mata D Crespo A (2011) Another example of cryptic diversity in lichen-forming fungi: the new species *Parmeliamayi* (Ascomycota: Parmeliaceae). Organisms Diversity & Evolution 11: 331–342. 10.1007/s13127-011-0060-4

[B48] Nelsen MP Chavez N Sackett-Hermann E Thell A Randlane T Divakar PK Rico VJ Lumbsch HT (2011) The cetrarioid core group revisited (Lecanorales: Parmeliaceae). Lichenologist 43: 537–551. 10.1017/S0024282911000508

[B49] Onut-Brännström I Johannesson H Tibell L (2018) *Thamnoliatundrae* sp. nov., a cryptic species and putative glacial relict. Lichenologist 50: 59–75 10.1017/S0024282917000615.

[B50] Orange A James PW White FJ (2001) Microchemical methods for the identification of lichens. London: British Lichen Society.

[B51] Otte V Esslinger TL Litterski B (2005) Global distribution of the European species of the lichen genus *Melanelia* Essl. Journal of Biogeography 32: 1221–1241. 10.1111/j.1365-2699.2005.01268.x

[B52] Palice Z Printzen C (2004) Genetic variability in tropical and temperate populations of *Trapeliopsisglaucolepidea*: evidence against long range dispersal in a lichen with disjunct distribution. Mycotaxon 90: 43–54.

[B53] Penn O Privman E Ashkenazy H Landan G Graur D Pupko T (2010) GUIDANCE: a web server for assessing alignment confidence scores. Nucleic Acids Research 38(Web Server issue): W23–W28. 10.1093/nar/gkq443 PMC2896199 20497997

[B54] Printzen C Ekman S (2003) Local population subdivision in the lichen *Cladoniasubcervicornis* as revealed by mitochondrial cytochrome oxidase subunit 1 intron sequences. Mycologia 95: 399–406. 10.1080/15572536.2004.11833084 21156628

[B55] Printzen C Ekman S Tønsberg T (2003) Phylogeography of *Cavernulariahultenii*: evidence for slow genetic drift in a widely disjunct lichen. Molecular Ecology 12: 1473–1486. 10.1046/j.1365-294X.2003.01812.x 12755876

[B56] Rambaut A (2012) FigTree v.1.4.2. http://tree.bio.ed.ac.uk/software/figtree/

[B57] Renner SS (2016) A return to Linnaeus’s focus on diagnosis, not description: The use of DNA characters in the formal naming of species. Systematic Biology 65(6): 1085–1095. 10.1093/sysbio/syw032 27146045

[B58] Rico VJ van den Boom PP Barrasa JM (2005) Morphology, chemistry and distribution of *Melaneliasorediella* (Parmeliaceae) and similar species in the Iberian Peninsula. Lichenologist 37: 199–215. 10.1017/S0024282905014830

[B59] Ronquist F Huelsenbeck JP (2003) MrBayes 3: Bayesian phylogenetic inference under mixed models. Bioinformatics 19: 1572–1574. 10.1093/bioinformatics/btg180 12912839

[B60] Rozas J Ferrer-Mata A Sánchez-DelBarrio JC Guirao-Rico S Librado P Ramos-Onsins SE Sánchez-Gracia A (2017) DnaSP 6: DNA Sequence Polymorphism Analysis of Large Datasets. Molecular Biology and Evolution 34: 3299–3302. 10.1093/molbev/msx248 29029172

[B61] Sela I Ashkenazy H Katoh K Pupko T (2015) GUIDANCE2: accurate detection of unreliable alignment regions accounting for the uncertainty of multiple parameters. Nucleic Acids Research 43(Web Server issue): W7–W14. 10.1093/nar/gkq443 PMC4489236 25883146

[B62] Spribille T Klug B Mayrhofer H (2011) A phylogenetic analysis of the boreal lichen *Mycoblastussanguinarius* (Mycoblastaceae, lichenized Ascomycota) reveals cryptic clades correlated with fatty acid profiles. Molecular Phylogenetics and Evolution 59: 603–614. 10.1016/j.ympev.2011.03.021 21443957 PMC3093615

[B63] Stamatakis A (2014) RAxML Version 8: A tool for phylogenetic analysis and post-analysis of large phylogenies. Bioinformatics 30: 1312–1313. 10.1093/bioinformatics/btu033 24451623 PMC3998144

[B64] Starosta J Svoboda D (2020) Genetic variability in the *Physconiamuscigena* group (Physciaceae, Ascomycota) in the Northern Hemisphere. Lichenologist 52: 305–317. 10.1017/S0024282920000134

[B65] Szczepańska K Kossowska M (2017) *Cetrariellacommixta* and the genus *Melanelia* (Parmeliaceae, Ascomycota) in Poland. Herzogia 30: 272–288. 10.13158/heia.30.1.2017.272

[B66] Szczepańska K Pruchniewicz D Sołtysiak J Kossowska M (2015) Lichen-forming fungi of the genus *Montanelia* in Poland and their potential distribution in Central Europe. Herzogia 28: 697–712. 10.13158/heia.28.2.2015.697

[B67] Szczepańska K Urbaniak J Śliwa L (2020) Taxonomic recognition of some species-level lineages circumscribed in nominal *Rhizoplacasubdiscrepans* s. lat. (Lecanoraceae, Ascomycota). PeerJ 8: e9555. 10.7717/peerj.9555 PMC7409781 32832264

[B68] Thell A (1995) A new position of the *Cetrariacommixta* group in *Melanelia* (Ascomycotina, Parmeliaceae). Nova Hedwigia 60: 407–422.

[B69] Thell A Crespo A Divakar PK Kärnefelt I Leavitt SD Lumbsch HT Seaward MRD (2012) A review of the lichen family Parmeliaceae – history, phylogeny and current taxonomy. Nordic Journal of Botany 30: 641–664. 10.1111/j.1756-1051.2012.00008.x

[B70] Werth S Sork VL (2008) Local genetic structure in a North American epiphytic lichen, *Ramalinamenziesii* (Ramalinaceae). American Journal of Botany 95: 568–576. 10.3732/ajb.2007024 21632383

[B71] Westberg M Kärnefelt I Thell A (2004) *Melaneliaagnata*, an overlooked species, new to Sweden. Graphis Scripta 16(1): 23–27.

[B72] Xu M Heidmarsson S Thorsteinsdottir M Eiriksson FF Omarsdottir S Olafsdottir ES (2017) DNA barcoding and LC-MS metabolite profiling of the lichen-forming genus *Melanelia*: Specimen identification and discrimination focusing on Icelandic taxa. PLоS ONE 12(5): e0178012. 10.1371/journal.pone.0178012 PMC5443556 28542495

